# The discovery of Lake *Hephaestus*, the youngest athalassohaline deep-sea formation on Earth

**DOI:** 10.1038/s41598-018-38444-z

**Published:** 2019-02-08

**Authors:** Violetta La Cono, Giovanni Bortoluzzi, Enzo Messina, Gina La Spada, Francesco Smedile, Laura Giuliano, Mireno Borghini, Christine Stumpp, Philippe Schmitt-Kopplin, Mourad Harir, William K. O’Neill, John E. Hallsworth, Michail Yakimov

**Affiliations:** 1 CNR, Institute for Coastal Marine Environment, Messina, 98122 Italy; 2 CNR, Institute for Marine Sciences, Bologna, 40129 Italy; 3 Mediterranean Science Commission (CIESM), MC, 98000 Monaco; 4 CNR, Institute for Marine Sciences, La Spezia, 19136 Italy; 5 Institute of Groundwater Ecology, Helmholtz Centre Munich, Neuherberg, 85764 Germany; 6 Institute of Hydraulics and Rural Water Management, University of Natural Resources and Life Sciences Vienna, Wien, 1190 Austria; 7 Research Unit Analytical BioGeoChemistry, Helmholtz Centre Munich, Neuherberg, 85764 Germany; 8 Technische Universität München, Lehrstuhl für Analytische Lebensmittelchemie, Freising, 85354 Germany; 9 Institute for Global Food Security, School of Biological Sciences, MBC, Queen’s University Belfast, Belfast, BT9 7BL Northern Ireland UK; 10 Institute of Living Systems, Immanuel Kant Baltic Federal University, Kaliningrad, 236016 Russia

**Keywords:** Marine biology, Microbial ecology, Astrobiology, Marine chemistry

## Abstract

Hydrated, magnesium-rich minerals and subglacial brines exist on the martian surface, so the habitability of high-Mg^2+^ environments on Earth has extraterrestrial (as well as terrestrial) implications. Here, we report the discovery of a MgCl_2_-dominated (4.72 M) brine lake on the floor of the Mediterranean Ridge that underlies a 3500-m water column, and name it Lake *Hephaestus*. Stable isotope analyses indicated that the *Hephaestus* brine is derived from interactions between ancient bishofite-enriched evaporites and subsurface fluids. Analyses of sediment pore waters indicated that the *Hephaestus* depression had contained the MgCl_2_ brine for a remarkably short period; only 700 years. Lake *Hephaestus* is, therefore, the youngest among currently known submarine athalassohaline brine lakes on Earth. Due to its biologically hostile properties (low water-activity and extreme chaotropicity), the *Hephaestus* brine is devoid of life. By contrast, the seawater-*Hephaestus* brine interface has been shown to act as refuge for extremely halophilic and magnesium-adapted stratified communities of microbes, even at MgCl_2_ concentrations that approach the water-activity limit for life (0.653).

## Introduction

Characterization of the martian surface, using both rovers and orbital technologies, has yielded evidence of hypersaline paleolakes on ancient Mars. They were widely distributed in time and space and likely an order of magnitude more saline than terrestrial seawater^1,2^. Recent radar data indicated the existence of a large body of hydrated salts/brines in a subglacial region of Mars’ southern ice cap^3^. The temperature under the ice cap, approximately 205 K (−68 °C), indicates that this putative lake is hypersaline^3^. Experimental and theoretical insights into weathering of martian basalt reveal that martian brines are likely dominated by magnesium and calcium (i.e. athalassohaline) and so qualitatively different from the sodium-dominated (thalassohaline) waters that are commonplace on Earth^3^. Considerable amounts of Mg^2+^ were discovered in the soil of the northern plains of Mars in the form of perchlorates and chlorides^4^. Furthermore, chloride salts have been found in widespread deposits on the southern Martian hemisphere^5^. At high concentrations, both perchlorate and chloride salts disrupt hydrogen bonding and strongly suppress the freezing point of water (down to 204-198 K). Martian perchlorates can form through photo-oxidation of chloride by oxide minerals and other mechanisms, both in its lithosphere and atmosphere^6^. Thus, the brine lake under the polar ice cap, which presumably formed under dark and high-pressure conditions, may contain high concentrations of unoxidized chloride salts. Some anaerobic and halophilic prokaryotes have a considerable tolerance to perchlorates^7,8^ so, assuming that temperatures occasionally reached more than 248 K (−25 °C), a subglacial brine lake may constitute a Mars Special Region where terrestrial organisms might replicate^9^.

Two deep-sea lakes located on Earth, on the seabed of the Mediterranean Ridge (>3,300 mbsl), were formed by deliquescence of hygroscopic bischofite (MgCl_2_•6H_2_O). These lakes are called *Discovery* and *Kryos* and are under pressure due to the overlying seawater, dark, anoxic and athalassohaline and, as such, may be considered analogues of the subglacial martian brines. The seawater-brine interface of each of these deep-sea lakes acts as a refuge for active microbial communities^10–12^. The biology and biophysics of these MgCl_2_-dominated systems has potential to inform planetary protection policy and upcoming space-exploration missions which focus on life detection. Whereas liquid water is essential for terrestrial life, the habitability of hydrological bodies also depends on the thermodynamic availability of this water, i.e., water activity (a_w_). Indeed, this parameter imposes sharply defined constraints on all types of organism. For a long time, the a_w_ limit for the most-extremely halophilic bacteria and archaea was considered to be 0.755 and it was thought that ionic and organic solutes imposed different biophysical limitations on microbial systems^13^. However, recent studies have demonstrated differentiation and cell division of halophilic bacteria and archaea at a_w_ values close to 0.600 in saline milieu^14^, and of ascomycete fungus, *Aspergillus penicillioides*, at 0.585 a_w_ in glycerol-supplemented media^15^. Extrapolations of experimental results have yielded a theoretical a_w_ minimum for extreme fungal xerophiles in the range 0.570–0.565^15^.

The *Discovery* and *Kryos* lakes are polyextreme environments, and there is a substantial thermodynamic distance across the water-activity scale between the point where life processes cease (0.585 water activity) and a_w_ values (<0.400) of these deep-sea brines^10,11^. In addition, MgCl_2_ concentrations of more than approx. 3.0 M have been shown to be beyond the limits of cellular tolerance, regardless of the domain of life^10,11,14,16,17^. Empirical determinations show that a 5 M MgCl_2_ solution has a chaotropic activity of 212 kJ g^−1^, which is more than twice that of a saturated solution of phenol^11^. The thin (≤3 m) interfaces between Mediterranean seawater and the underlying MgCl_2_ brines of *Discovery* and *Kryos* provide a unique biophysical environment to study the limits of life at high magnesium. In the *Discovery* brine, magnesium chloride is close to purity^11^. In the *Kryos* system, there are significant concentrations of ions that are known to stabilize biomacromolecules of cellular systems (i.e. they mitigate chaotropicity)^10^. These are termed ‘kosmotropes’ and include sulfate ions as well as Na^+^, though the latter is less kosmotropic than the former^16,18^. Previously, we demonstrated that the upper concentration of MgCl_2_, that permits life within the seawater-*Discovery* interface, is about 2.3 M^11^. Lake *Kryos* contains higher concentrations of Na^+^ and SO_4_^2−^ ions and, accordingly, we were able to recover mRNA molecules - indicators of metabolically active cells - from the 2.27–3.03 M MgCl_2_ layer (a_w_ 0.747–0.631), thereby expanding the recognized chaotropicity window-of-life^10^.

Here, we report the discovery of a third MgCl_2_-dominated deep-sea formation, and name it Lake *Hephaestus*. We characterize key aspects of its geological origin, geochemistry and microbial ecology and reveal that *Hephaestus* is the youngest athalassohaline lake on Earth so far.

## Results and Discussion

### Discovery of the athalassohaline Lake *Hephaestus*

**Figure 1:**
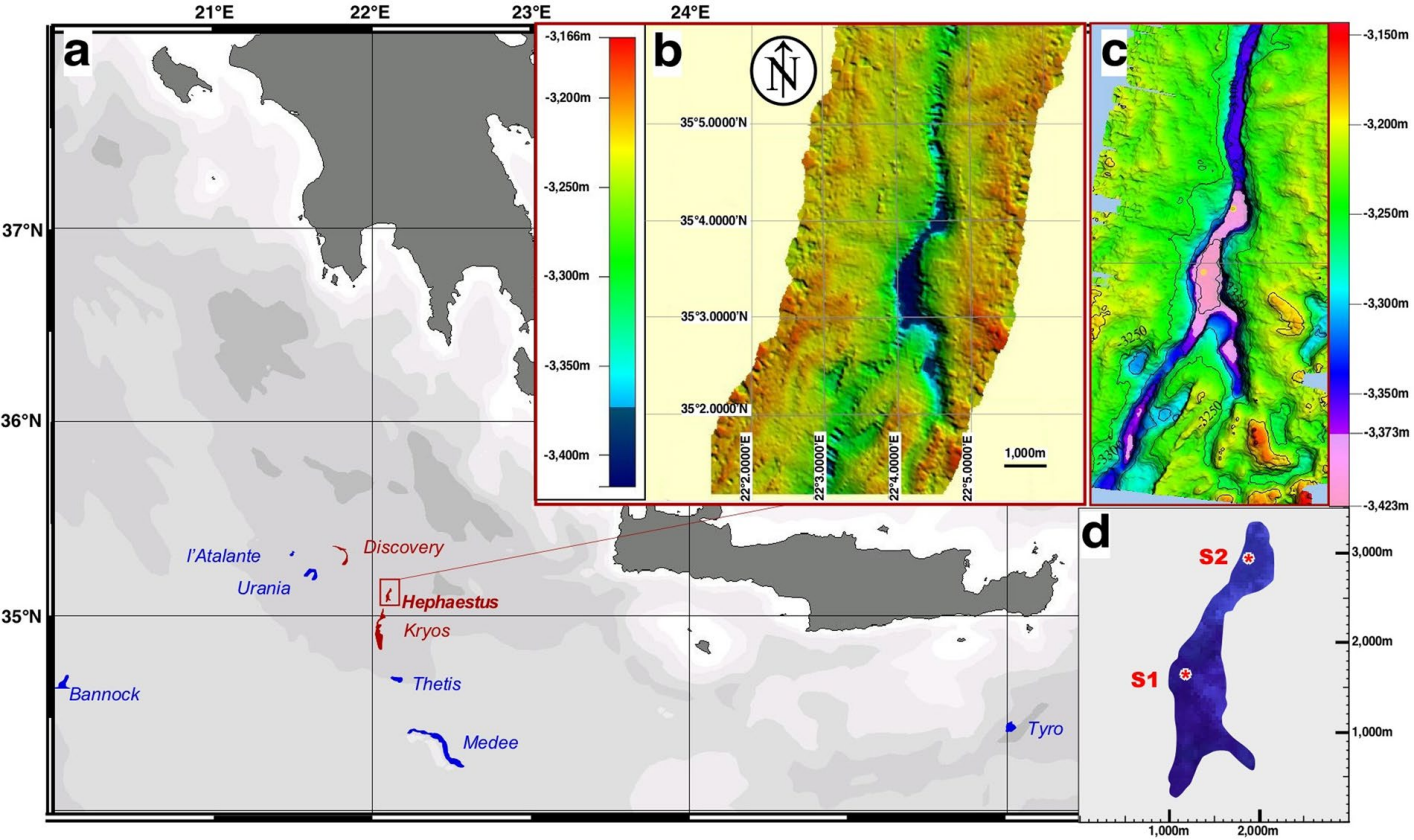
Locations of all nine deep-sea brine lakes currently known to exist within the Mediterranean Ridge. (**a**–**c**) MgCl_2_-filled lakes are shown in red, while thalassohaline lakes are shown in blue. Swath bathymetry shows images of the Lake *Hephaestus* area with 10-m (**b**) and 5-m resolution (**c**). For (**c**), depressions of ≥ 3,373 m in depth are shown in pink. (**d**) Locations of sampling site S1 (35°03.32 N; 22°04.11E) and S2 (35°03.99 N; 22°04.55E). Multibeam swath bathymetry (**b**,**c**) was obtained by echo-sounding and processed with NEPTUNE, CARIS and GMT packages^19^.

The first oceanographic, geochemical and microbiological characterizations of Lake *Hephaestus* were performed, on the research vessel *ROV Urania*, during two consecutive cruises DEEP_PRESSURE (9–22/10/2013) and SALINE (21/10-03/11/2014). Moving three nautical miles *NNE* from Lake *Kryos*, a steep fracture was discovered and mapped by 3.5 Chirp kHz swath-bathymetry profiling (SBP). The fracture is 10 km in length, serpentine in shape, oriented along a *N-S* axis, and has an arm on the *SE* side. A basin was situated in its central part, with a maximum depth of 3,423 m, i.e. 120–150 m beneath the surrounding seabed. SBP produced a sharp crisp line over the basin and hinted at the existence of a brine lake, that we named *Hephaestus*. This was confirmed by direct conductivity-temperature-dissolved oxygen (CTD) profiling, followed by retrieval of samples of the brine. Using the SBP data and determination of brine densities, we determined the exact depths and then used pressure data from the CTD casts to map the depression (Fig. 1).

Since all conventional online CTD sensors are calibrated for seawater, they are not fully functional in athalassohaline environments, that are characterized by ratios of mono- and divalent cations different from those of seawater. Nevertheless, we were able to map the exact location of the seawater-*Hephaestus* brine interface and the brine itself (Supplementary Fig. 1), although chemical analysis of fractionated interface samples and brine were performed in on-land laboratory. Lake *Hephaestus* has its 3 m-thick seawater–brine interface at 3,373 m and occupies the steep, narrow basin that is approximately 3-km long and 0.7-km wide. The depth of the brine, which forms the lake, is ≤50 m. The temperature measured at the seawater–brine interface was 14.00 °C and increased linearly within the brine depth to 15.26 °C, close to the seabed. The *S-E* arm appears to be separated from the lake’s main body by a 10–20 m high section of the seabed as evidenced by swath-bathymetry profiling and lack of sharp crisp SBR line. The name *Hephaestus* came from the Greek god of fire and volcanoes who spent his childhood in the Mediterranean deep.

The discovery of this deep-sea lake, just few kilometres from Lake *Kryos*, was unexpected. We found it noteworthy that the upper surface of the *Hephaestus* brine is some 36 m lower than that of Lake *Kryos*. Chemical characterization of *Hephaestus* brine confirmed that these two lakes constitute separate hydrologic milieu, although it shares the athalassohaline character of the *Kryos* and *Discovery* brines; it contains MgCl_2_ close to saturation (4.72 M). In terms of the major ions, *Hephaestus* is intermediate between the *Kryos* and *Discovery* brines and all three lakes are highly chaotropic (Table 1).

**Table 1:** Major ion composition and other parameters of MgCl_2_-dominated, deep-sea brines from the Mediterranean Ridge.

	*Hephaestus* brine	*Kryos* brine	*Discovery* brine	Mediterranean seawater
*Major ions, mmol kg* ^*−*1^				
Na^+^	93	125	84	540
K^+^	28	80	20	12
Mg^++^	4,720	4,380	5,150	61
Ca^++^	2	1	1	12
Cl^−^	9,120	9,043	10,150	630
SO_4_^−^	203	320	110	33
Br^−^	78	70	110	1
*Principal parameters*				
Practical salinity units, PSU	480	470	510	39
δ^18^O, ‰	−3.06 ± 0.22	ND	−2.39 ± 0.79	0.97 ± 0.68
δ^2^H, ‰	−16.5 ± 0.4	ND	−17.9 ± 1.5	5.1 ± 5.7
pH	5.0	4.5	5.4	7.85
Density	1.32	1.32	1.33	1.0
Temperature, °C	15.3	14.6	14.5	13.7
Water activity, a_w_	**0.395**	**0.399**	**0.382**	0.980
Brine, mbsl	>3,373	>3,337	>3,580	NA
Maximum depth	≈50 m	≈160 m	≈50 m	NA
Surface area, km^2^	≈1.7	≈25	≈7.5	NA

ND, not determined.

NA, not applicable.

### Origin and age determination of the *Hephaestus* lake

*Hephaestus* is likely to have originated the same way as the *Kryos* and *Discovery* brines that formed by dissolution of bischofite containing 8 g kg^−1^ Br^19^. This Br concentration is typically found in bischofite, which precipitates from brine containing higher Br concentration (12 g kg^−1^) that occurs when seawater is evaporated to less than 1% of its volume^20^. Thus, the discovery of the *Hephaestus* brine (along with those of *Kryos* and *Discovery*) reinforces the existing body of evidence that the Eastern Mediterranean evaporated close to dryness during the Messinian salinity crisis, 5–6 million years ago^20–22^. Analyses of stable isotope δ^18^O-H_2_O and δ^2^H-H_2_O ratios for the *Hephaestus* and *Discovery* brines can shed light on the origins of these lakes. Both these ratios are depleted relative to that of Mediterranean seawater (Table 1). This indicates water of meteoric origin of the past or connate water; *i.e*. modern seawater was not the main water source of these deep-sea brines. Bischofite is a highly hygroscopic late-stage evaporitic substance that easily deliquesces upon contact with interstitial water, so subsurface deposits of bischofite are readily converted to subsurface brine lenses.

**Figure 2:**
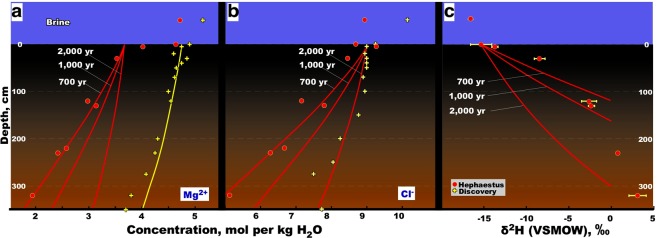
Hydrochemical and stable isotope analysis of *Hephaestus* sediments. (**a**–**c**) Concentrations of Mg^2+^ (**a**), Cl^−^ (**b**) and δD (versus Vienna standard mean ocean water [VSMOW]) (**c**) in the pore water of a 3.2-m long sediment core. The bars represent δD standard deviation of measurements of brine and pore water samples made on three replicate assays. Concentrations of Mg^2+^, Cl^−^ in *Discovery* sediments^20^ are shown for comparison. All model curves (see Methods) for Mg^2+^ and Cl^−^ were produced using the following parameters: effective sediment diffusion coefficient (*D*_*s*_), 2.4 × 10^−10^ m^2^ s^−1^; advection rate (*V*_*s*_), 9.51 × 10^−11^ m s^−1^; timing (*t*), 2.2 × 10^10^ s (≈700 years), 31.55 × 10^9^ s (≈1,000 years), 63.1 × 10^9^ s (≈2,000 years) for *Hephaestus*, 2,000 years for *Discovery* (Cl^−^ is overlapped). Model curves for δD share the same parameters and timings, except for sediment diffusion coefficient *D*_*s*_ set to 2.96 × 10^−10^ m^2^ s^−1^. Error bars reflect the standard deviation.

Whereas it is not known for how long dissolved bischofite remained in this way as interstitial pools, it is most likely that the brine was extruded to the surface of seabed through vents or fractures created by the tectonic activity and/or by compaction-induced advection that is commonplace at the Mediterranean Ridge^22,23^. The extremely viscous and high-density brine then settled in the *Hephaestus* basin. Analysis of a 3.2-m sediment core from the deepest part of the brine lake (sampling site S1) indicated that Mg^2+^ and Cl^−^ concentrations decrease sharply with sediment depth. Conversely, and in accordance with this finding, δH^2^-H_2_O values increased, and approach those of Mediterranean seawater, with depth (Fig. 2). However, δ^18^O-H_2_O values in the pore water of the sediments were enriched (3–8.2‰) indicating interactions of pore water with mineral water^24^. Using the same one-dimensional diffusive transport model^20^, we calculated the diffusion time that would be required to obtain concentrations of Mg^2+^ and Cl^−^ that were empirically determined. Different time intervals were applied in the modelling (Fig. 2 and Supplementary Methods). The results obtained indicate that the *Hephaestus* surface sediments have been in contact with MgCl_2_ brines for a period of about 700 years. Lake *Hephaestus* therefore, formed 1,300 years later than Lake *Discovery* which was previously considered the most recent of the Mediterranean deep-sea brine lakes, some of which are 35,000–180,000 years old^20,22^. So *Hephaestus*, one of the three most saline athalassohaline formations known to exist in submarine locations, is the youngest such brine lake on Earth.

### Characterization of dissolved organic matter in the *Hephaestus* brine and seawater-brine interface

**Figure 3:**
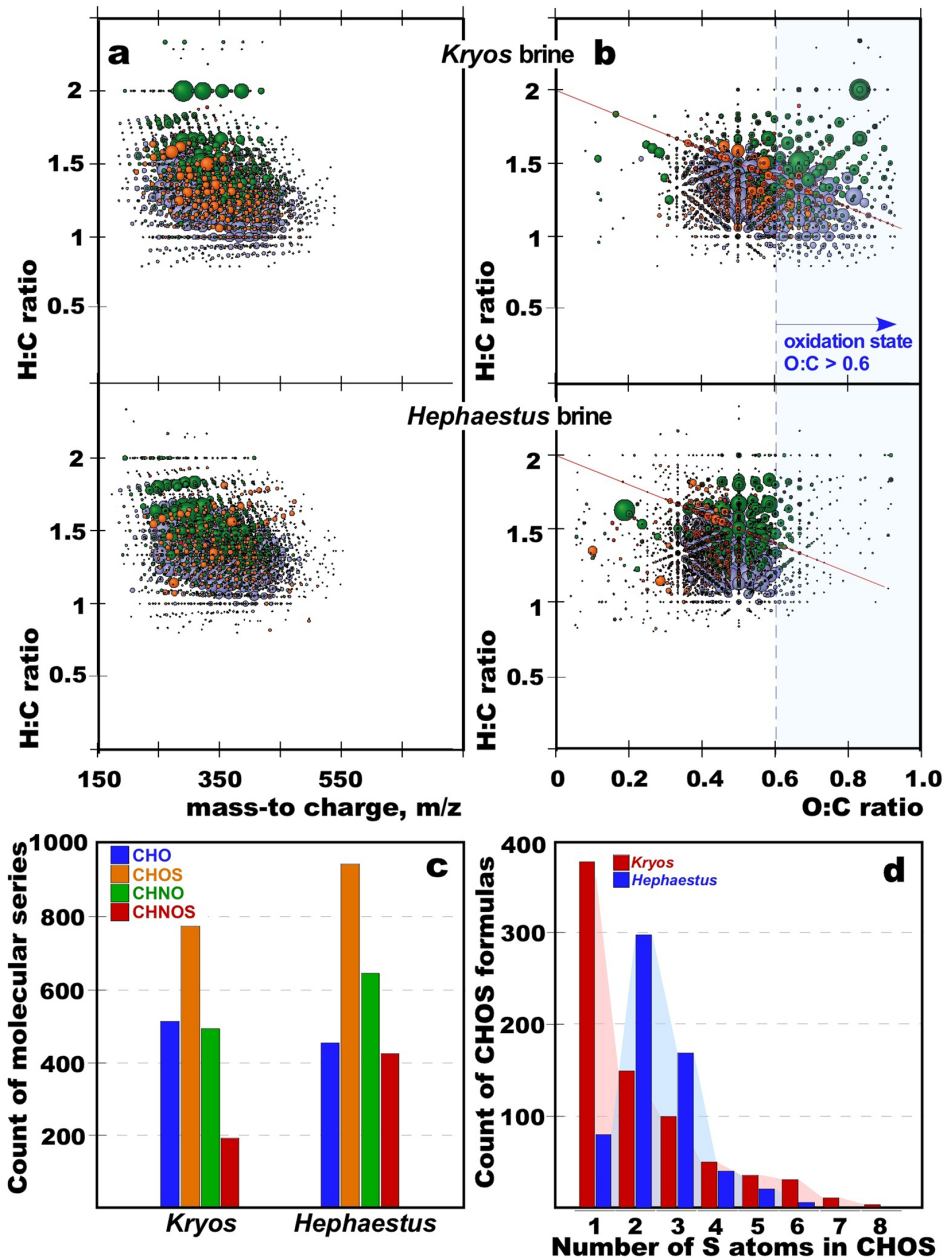
Ultra-high resolution negative ICR-MS mass spectrometry of the *Kryos* and *Hephaestus* brine DOM. (**a**) Mass-to-charge versus H:C ratio, showing hundreds of low molecular weight organic compounds. (**b**) van Krevelen diagrams, illustrating remarkable depletion of highly oxygenated (O:C > 0.6) molecular series in the *Hephaestus* brine DOM. The red line refers to the compositional range of carboxyl-rich alicyclic materials. Bubble colour code for molecular formula series with C, H, O, N and S combinations are defined as follow: CHO (blue), CHOS (green), CHON (orange) and CHONS (red). Bubble areas indicate relative mass peak intensity of each assigned mass ion. (**c**) Counts of elemental compositions of assigned molecular formulas CHO, CHNO, CHOS and CHNOS. (**d**) Counts of sulphur atoms in CHOS-containing compounds.

Using ultra-high resolution ion cyclotron resonance Fourier transform mass spectrometry (ICR-FT-MS), the analysis of dissolved organic matter (DOM) in the *Hephaestus* brine showed thousands of relatively small organic compounds of a mean molecular weight (<500 amu). This DOM is almost certainly the remains of complex organic materials that have been highly processed by saprotrophic activity (Fig. 3a,b), as demonstrated for the lake *Kryos* brine^10^. Relative abundances of the primary compounds (CHO, CHNO, CHOS and CHNOS) within the total DOM from the *Hephaestus* brine indicates the dominance of sulfurized assigned molecular series (Fig. 3c), which indicates the importance of sulfur chemistry in this extreme and sulfide-rich brine. The ratios of CHOS/CHO (2.11) and CHNOS/CHNO (0.66) in the *Hephaestus* brine are similar to those in the *Kryos* brine (1.55 and 0.43, respectively; (Fig. 3c)), both of which substantially differ from marine DOM ratios (0.25 and 0.14^24^) and are rather reminiscent of those of compounds, produced abiotically from CHO and CHNO compounds by reactive sulfur species (1.66 and 1.12^25^). The van Krevelen diagrams show that the *Hephaestus* brine DOM has a higher chemical diversity and more pronounced aliphaticity and sulfurization than that of *Kryos* brine (Fig. 3b). At the same time the *Hephaestus* brine DOM exhibits remarkable depletion of highly oxygenated (O/C ratio >0.6) organic compounds, which are characteristic of biologically active environments, including deep seawater^26^. Since this deep-sea lake is an open hydrological formation, its surface layer is inevitably influenced by the overlying seawater column, which is in line with the ICR-FT-MS analysis of the seawater-*Hephaestus* interface (layer of 3.03–4.11 M Mg^2+^), revealing a notable presence of highly oxygenated (O/C ratio >0.6) molecular series (Supplementary Fig. 3). Given that Lake *Kryos* is older than *Hephaestus* and therefore is interacting with seawater for longer time, the presence of highly oxygenated organic compounds in the lifeless *Kryos* brine was not unexpected (Supplementary Fig. 2). Other data also indicate that the Lake *Hephaestus* brine is less influenced by the surficial mixing with oxygenated seawater than that of Lake *Kryos*. ICR-FT-MS comparative analysis of the *Hephaestus* brine and across its seawater-brine interface revealed that the majority of identified organic compounds (58%) were unique to either the brine or interface (Supplementary Fig. 4). By contrast, for the *Kryos* system, only one third of such organic mass ions (37%) were determined as unique to either the brine or interface (Supplementary Fig. 5). Elevated presence of reduced and polysulfuric organic signatures in the *Hephaestus* brine DOM as well as the highest abundance of the low oxygenated heteroatomic CHNO and CHOS compounds (Supplementary Fig. 6), are consistent with the hypothesis that Lake *Hephaestus* was formed upon deliquescence of bischofite-enriched evaporitiic deposits with highly reduced euxinic subsurface fluids, rather than with oxygenated deep seawater.

### High-resolution sampling of the seawater-*Hephaestus* brine interface

**Figure 4:**
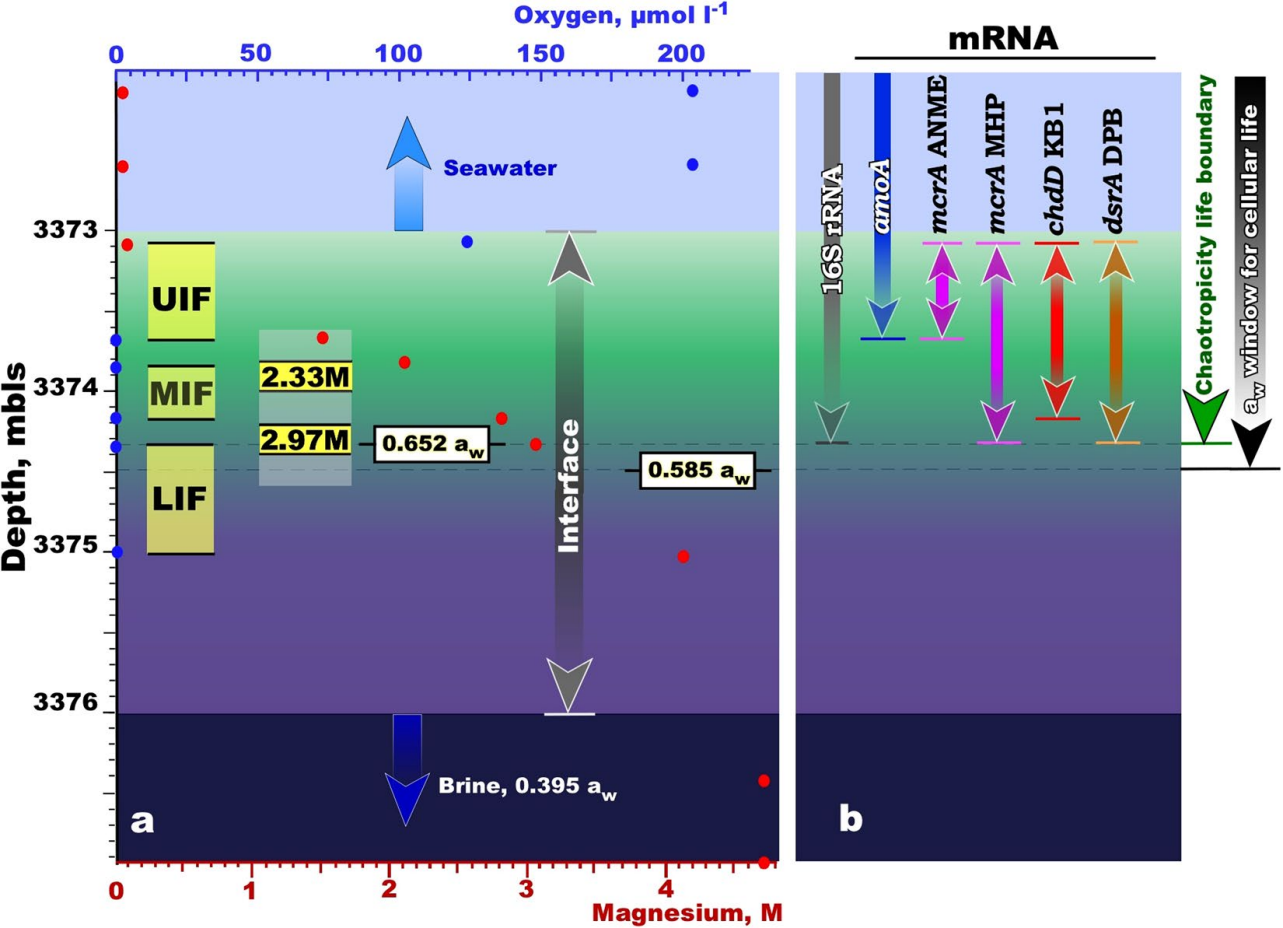
Overview of high-resolution sampling, depth profile of magnesium and oxygen concentration and the RNA stratification through Lake *Hephaestus* interface. (**a**) Profiles of geochemical markers (Mg^2+^ and O_2_) through Lake *Hephaestus* and location of the established boundary for xerophilic cellular life (a_w_ = 0.585). Positioning of the interface and brine was established by CTD profiling (Supplementary Fig. 1). The layers of the interface were collected for molecular and chemical analyses during two consecutive cruises and highlighted in grey (DEEPPRESSURE_2013) and yellow (SALINE_2014). Abbreviation used: UIF, upper interface; MIF, middle interface; LIF, low interface. Data points are mean ± standard error (*n* = 3). (**b**) Nucleic acids recovery from seawater, interface and brine. Types of mRNA gene transcripts are discussed through the text.

There is a sharp halocline separating the *Hephaestus* brine from the overlaying seawater. The Mg^2+^ gradient of this ∼3.0-m interface ranges from 70 mM at the top to 4,720 mM where it meets the underlying brine (Fig. 4a). Using our previous approach for a_w_ measurements of MgCl_2_ solutions, synthetic brines, and *Discovery* and *Kryos* interfaces^10,11^, we measured the a_w_ across the seawater-*Hephaestus* brine interface, as well as that of the brine itself (Fig. 4a). A value of 0.585 lies more or less midway down the *Hephaestus* interface, indicating that only the upper half of the interface corresponds to the currently recognized a_w_ window for microbial function. After conducting a high-resolution sampling (Supplementary Fig. 7), three fractions of the interface were chosen for further biomolecular analyses: the upmost subsample, which had Mg^2+^ concentrations from 70 mM to 1,500 mM (named as upper interface; UIF), the median subsample, which had Mg^2+^ concentrations from 2,080 mM to 2,800 mM (mid interface; MIF), and the lower subsample, which had Mg^2+^ concentrations from 3,050 mM to 4,120 mM (lower interface; LIF). Since detection of active microbial life below the currently recognized a_w_ window was highly improbable, the bottom-most fraction of the interface from 4,120 mM to 4,720 mM was not analysed.

Environmental DNA and, to a lesser extent, rRNA can be stable under the chaotropic, low a_w_, ionic conditions of MgCl_2_-saturated deep-sea brines and, thus, cannot be relied on as a marker of microbial activities^10,11,27^ (Supplementary Fig. 8). We therefore extracted total RNA from the recovered gradient to survey the distribution of ribosome-containing and metabolically active prokaryotes. Further comparative analysis of ribosomal RNA (rRNA) and the much less stable messenger RNA (mRNA) transcripts were performed with total complementary DNA (cDNA), obtained by reverse transcription with hexa-random primers, followed by polymerase chain reaction (PCR) with specific primers (Supplementary Table 1). No cDNA transcripts of the target mRNA transcripts were obtained in the LIF fraction, indicating the absence of viable microbes due to extreme harshness of chaotropic environments at Mg^2+^ concentrations >3.05 M. Since there was a gap in the Mg^2+^ concentrations between the UIF and MIF fractions, in order to achieve continuity of the gradient and make precise determinations of the boundary conditions for microbial habitability, the middle section of the *Hephaestus* interface was carefully collected without perturbation or mixing during the subsequent SALINE_2014 cruise. The sample was retrieved from station S1, using a newly developed upward sampling strategy (Supplementary Fig. 7). Immediately after cast recovery, initial measurements of salinities of the top- and bottom-most content of Niskin bottles were performed. The bottles exhibiting equivalent range of salinities were carefully fractionated anaerobically and the 2,330 mM-Mg^2+^ and 2,970 mM-Mg^2+^ subsamples (Fig. 4a) were used for a series of comparative analyses.

### Identification of the boundary for active microbial life within the seawater-*Hephaestus* brine interface

**Figure 5:**
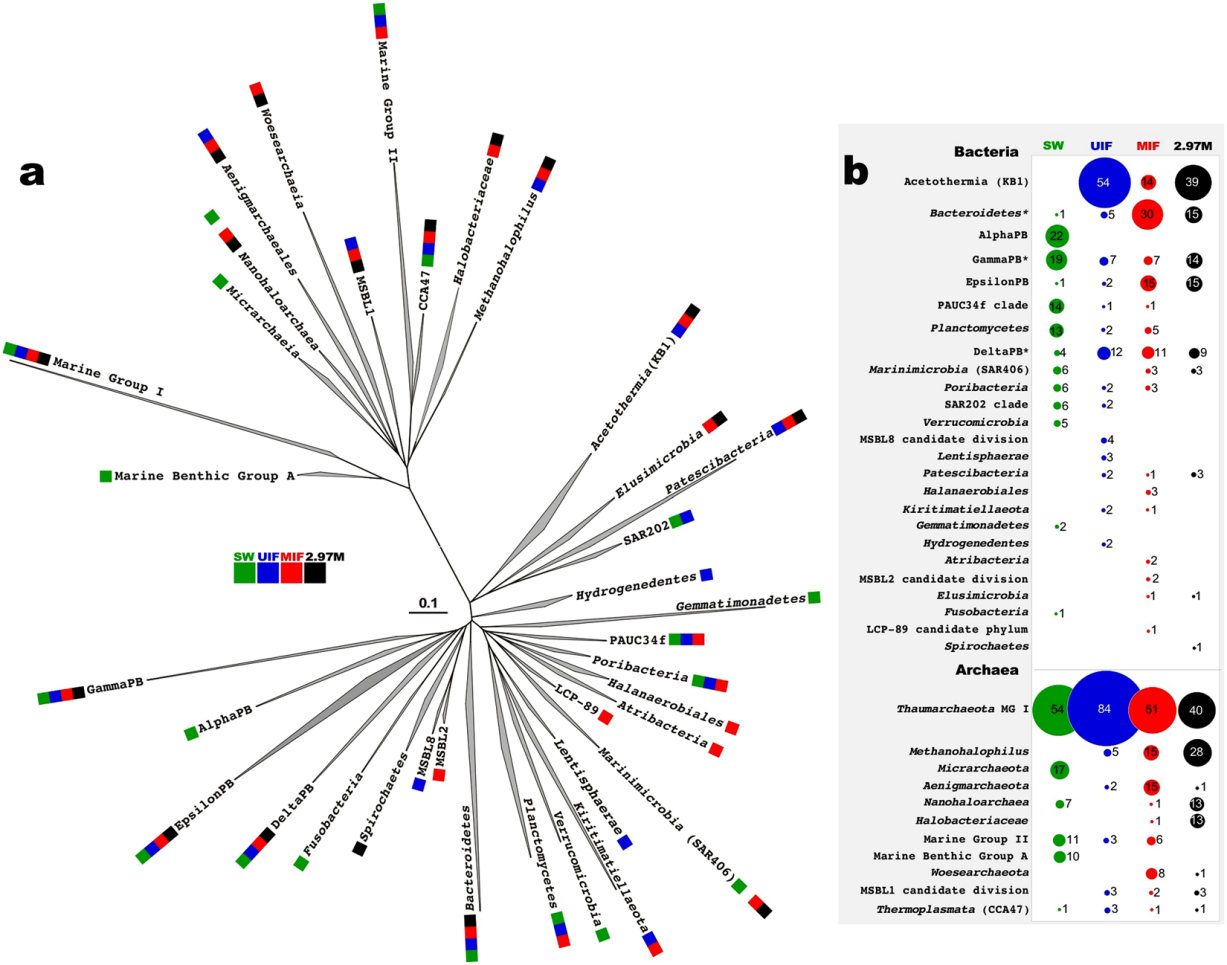
Stratification of microbial community, inhabiting various layers of the *Hephaestus* interface. (**a**) 16S rRNA Phylogenetic tree of phylogenetic groups, recovered from different compartments of the *Hephaestus* interface and the overlaying seawater. (**b**) Relative abundance, given as a percentage of all analysed clones from corresponding libraries. Phylogenetic tree was produced using ARB software and the updated SILVA database (May, 2018). Genus-level attribution of *Hephaestus* clones affiliated to three bacterial taxa, highlighted by asterisks, is given in Supplementary Table 2. Abbreviation used: 2.97 M, the subsample of low interface, collected in 2014; MIF, middle interface; SW, seawater sample, collected at 3,372 mbsl (one meter above the Hephaestus interface); UIF, upper interface.

Assessments of phylogenetic diversity, using the recovered 16S rRNA, revealed the existence of a stratified indigenous prokaryotic community in the seawater-*Hephaestus* brine interface up to 2,970 mM MgCl_2_ (Fig. 5, Supplementary Table 2). The 2,330 mM subsample exhibited a similar prokaryotic community to the MIF fraction that had been collected one year earlier. We therefore used the MIF 16S rRNA diversity (but not mRNA diversity) for further microbial diversity studies. The existence of layer-specific taxonomic groups provided confirmation that neither reciprocal mixing nor seawater contamination had been occurred during recovery and processing of interface samples. Obtained insight into phylogenetic diversity was used as a proxy for further monitoring of mRNA transcripts of genes involved in the seminal ecophysiological processes for the four most prevalent taxa in the deepest rRNA-containing subsample 2,970 mM (Fig. 5). These were: ammonium-oxidizing Marine Group I *Thaumarchaeota*; sulfate-reducing *Deltaproteobacteria*; methanotrophic and methanogenic (*Methanohalophilus* group) *Euryarchaeota* and the acetogenic members of KB1 group, recently attributed to *Acetothermia*^28^. In addition to gene markers that we used in an earlier study of the *Kryos* interface^10^ (Supplementary Table 1), the Wood-Ljungdahl pathway gene encoding for acetyl-CoA decarbonylase/synthase complex subunit delta (*cdh*D) was analysed to monitor the stratification of the KB1 group.

Determinations of mRNA-possessing microbes yielded a number of findings in relation to the ecology of the *Hephaestus* system. Firstly, metabolically active members of Marine Group I *Thaumarchaeota*, monitored by *amo*A transcripts, do not penetrate deeper than UIF layer (Supplementary Fig. 9). This also applied to the methanotrophic euryarchaeota unambiguously attributed to the ANME-1 Candidate Division, monitored by transcripts of methyl-coenzyme M reductase (*mcrA*), a gene involved in the first step of anaerobic activation of methane^29^ (Fig. 4b and Supplementary Fig. 10). Thus, since neither *amoA* nor methanotrophic homolog of mcrA transcripts were detected in deeper layers of the *Kryos* interface, it is plausible that both ammonium oxidation and anaerobic methane oxidation is limited by Mg^2+^ concentrations of >1,500 mM. Secondly, the *mcr*A transcripts, indicative of active methanogenic euryarchaeota of *Methanohalophilus* group, were undetectable at >2.97 M Mg^2+^ (Fig. 4b and Supplementary Fig. 10). Thirdly, we found no evidence of the *cdh*D transcripts in the interface deeper than 2,800 mM Mg^2+^ layer (Fig. 4b and Supplementary Fig. 11), despite the recovery of KB1 group (*Acetothermia*) 16S rRNA from the 2,970 mM MgCl_2_ layer. Fourthly, sulfate respiration, monitored according to presence of *dsr*AB transcripts, was (like methanogenesis) active only up to and including a Mg^2+^ concentration of 2,970 mM (Fig. 4b and Supplementary Fig. 12). As is the case for methanogenic *mcr*A, the *dsr*A sequences from the *Hephaestus* brine are deeply branched and clustered exclusively with sequences recovered from Lakes *Discovery* and *Kryos* or some other Mediterranean deep-sea brine lakes.

Despite the juvenility of Lake *Hephaestus*, it is clear that layer-specific microbial communities inhabit its upper interface. Furthermore, they are phylogenetically distinct from microbial populations of the overlying seawater. In relation to 16S rRNA phylogeny, and even more so for mRNA phylogeny, this community resembled those of the seawater-brine interfaces of *Discovery* and *Kryos*^10,11^. Collectively, these data allude to the existence of hitherto uncharacterized hyperhalophiles, adapted to resist the chaotropicity of MgCl_2_ and capable of metabolic activity under harshly athalassohaline conditions. As it discussed above, the bischofite and subsurface bischofite-derived brine from which Lake *Hephaestus* is made had already existed for a considerable time prior to entering the *Hephaestus* basin.

### The *Hephaestus* interface ecosystem: implication for astrobiology

The putative subglacial brine lake on Mars^3^, which is likely dominated by divalent cations, highly chaotropic and characterized by a low water activity, may be uninhabitable for terrestrial microbes, even at metabolically permissive temperatures. Aside from the lower temperature of the putative martian brine system, all athalassohaline Mediterranean deep-sea brine lakes share similar characteristics; they are anoxic, extremely salty, chaotropic and have high pressure. Being hostile to life, these lakes nevertheless provide biophysically and ecologically unique habitats for microbial ecosystems within their seawater-brine interfaces. The relatively youthful ecosystem of the *Hephaestus* interface offers a unique comparator system to those of the *Kryos* and *Discovery* interfaces. However, the Mg^2+^ concentration-limit for life within the seawater-*Hephaestus* interface, and the ion composition, exhibit intermediate characteristics relative to those of the latter indicating that biophysical parameters (rather than age) act as the key determinants of athalassohaline-brine ecology. High-resolution sampling of the *Hephaestus* interface and subsequent bio-molecular analyses confirm that there are no active microbial communities below the 2,970 mM Mg^2+^ layer. The empirically determined a_w_ value for this interface layer is 0.653, which is between the limits of life established for the *Discovery* and *Kryos* interfaces (0.790 and 0.631, respectively)^10,11^. Using constraints of fluid chemistry and saline mineralogy, the calculated a_w_ values for Meridiani Planum and other martian environments where salts precipitated from martian brines is ≤0.785^2^. Although these values are much below the levels of salinity tolerated by majority of known terrestrial organisms, they are thermodynamically mid-range^30^ and are comparable to, or even higher than the limits of life, estimated for *Discovery*, *Hephaestus* and *Kryos*.

Regarding the extremely low temperature of the putative martian subglacial lake, one more thought is worth consideration. Diverse lines of evidence, from known constraints on biotechnological processes to *in-vitro* studies of cellular stress metabolism and biophysics, show that chaotropic substances such as MgCl_2_ can be beneficial at low temperatures (those below 10 °C, and most especially sub-zero temperatures). Chaotropic activities impact biomacromolecules entropically by enhancing their flexibility, which is sufficient to reduce the temperature minimum for growth and metabolic activity of psychrophilic microbes^31–33^. Whereas such studies have yet to be carried out in the context of the *in-situ* ecology of halophiles, or at temperatures as low as −20 °C^32^, this biophysical phenomenon is likely to be universal. Therefore, the possibility remains that on moons or other planetary bodies, which are colder than Earth, high concentration of MgCl_2_ (or other chaotropic salts) can facilitate the habitability of their aqueous milieux. In relation to the planned space-exploration missions, we eagerly await the findings of those that will focus on life detection in the cold magnesium-rich subglacial systems, recently evidenced on Mars^3^.

## Methods

### Oceanographic characterization of the *Hephaestus* basin and high-resolution sampling

The target area was investigated with the hull mounted 16 transducer Benthos 3.5 KHz Chirp SBP. Multibeam swath bathymetry was obtained by the Kongsberg-Simrad EM-302 echosounding and processed with NEPTUNE, CARIS and GMT packages^19^. Samples of the *Hephaestus* Lake were collected using 18 × 12 liter Niskin bottles housed on a rosette (General Oceanics, Inc., Miami, FL, USA) equipped with SBE-911plus conductivity-temperature-depth (CTD) sensors (Sea-Bird Electronics, Inc., Bellevue, WA, USA). The interface was captured and fractionated as described elsewhere^10,34,35^ with slight modifications to perform a high-resolution sampling (Supplementary Fig. 7). Samples for determining major ion concentrations (20–100 ml) were collected in dark-polyethylene (DPE) vials and stored at room temperature prior the chemical analyses^35^. Dissolved anions and cations were quantified in diluted interface, brine and sediment pore water samples by ion chromatography using a coupled Dionex ICS 1100 system equipped with a AS4A 4 × 250 mm and a CS12 A 4 × 250 mm column. Pore water (2 ml) from the sediment cores was sampled using Rhizon samplers (Rhison SMS, Rhisophere Research Products).

### Stable isotopes of oxygen and hydrogen in brine and sediment pore water

Stable isotopes of oxygen and hydrogen (^18^O/^16^O and ^2^H/^1^H) were analyzed in brine and sediment pore water samples. Water from sediment samples was extracted via cryogenic extraction (T = 105 °C; the extraction was completed after 105–120 minutes)^36^. The extracted pore water samples and water samples from *Hephaestus* and the Mediterranean Sea were analysed on a pyrolysis-IRMS system containing a reactor filled with “glassy carbon” granulate (2,000−3,150 μm) and Ni-coated carbon (IVA Analyzentechnik, Meerbusch, Germany) at 1480 °C. After pyrolysis of H_2_O, CO and H_2_ were separated at 95 °C and subsequently transferred to a Finnigan MAT 253 isotope ratio mass spectrometer (Thermo Fisher Scientific, Bremen, Germany). Results are reported in the delta notation giving its deviation of concentration in parts per thousand from the Vienna Standard Mean Ocean Water (V-SMOW).

### Diffusion model calculation

Diffusion model curves were made for Mg^2+^, Cl^−^ and ^2^H concentration profile samples into pore water from brine in order to estimate the approximate age of *Hephaestus* sediments. Pore water profiles calculated for Discovery brine^20^ were also used for comparison. According to the Fick’s second law:

$$\frac{\partial C}{\partial t}={D}_{s}\frac{{\partial }^{2}C}{\partial {z}^{2}}-{{V}}_{{s}}\frac{\partial C}{\partial z}$$

where *D*_*s*_ is the (effective) sediment diffusion coefficient (in m^2^ s^−1^) for *in situ* conditions, corrected for tortuosity effect, *V*_*s*_ is the average linear pore water velocity (advection rate in m s^−1^), *C* is the *δD* concentration in water, *z* is the depth (in m), and *t* is the time (in s), we have modelled all curves shown in Fig. 2 by using the following analytical solution (eq. 21 in [Shackelford, 1991]):

$$C={C}_{o}\frac{1}{2}[{\rm{efrc}}(\frac{z-{V}_{s}t}{2\sqrt{{D}_{h}t/{R}_{d}}})+\exp (\frac{{V}_{s}z}{{D}_{h}}){\rm{erfc}}(\frac{z+{V}_{s}t}{2\sqrt{{D}_{h}t/{R}_{d}}})]$$

where *C*_0_ is the (average) initial concentration in brine with following boundary condition: *z* ≤ 0, *t* ≥ 0, and *D*_*h*_ ≈ *D*_*s*_ when *R*_*d*_ = 1 (nonreactive tracer, such as Cl^−^). *D*_*h*_ is the coefficient of hydrodynamic dispersion, *R*_*d*_ is the retardation factor, and *erfc* is the complementary error function. For simulations, we used values of *V*_*s*_ and *D*_*s*_ for Cl^−^ and Mg^−^ given in (Wallmann *et al*., 1997). We adapted diffusion coefficients of *δD* in free water to effective sediment diffusion coefficients for these extreme environmental conditions by the ratio of *D*_*s*_ for chloride ions^20^ and diffusion coefficients of Cl^−^ in free water^37^.

### Extraction of DOM and ultra-high resolution mass spectrometry (FT-ICR MS)

Untreated brine samples (200 ml) were filtered through pre-combusted Whatman GF/F glass fiber filters. The pH was adjusted to 2.0 by using high purity grade formic acid (98%). Solid-phase extraction (SPE) was followed using Agilent Bond Elut PPL SPE cartridges filled with highly functionalized styrene-divinylbenzene (SDVB) polymer that has been modified with a proprietary non-polar surface. The SPE cartridge was activated using methanol (Sigma-Aldrich Chromasolv LC-MS grade methanol), washed with acidified (pH 2.0) high purity water (Sigma-Aldrich Chromasolv LC-MS grade water). Then, the acidified sample was gravity-fed through the SPE cartridge. The cartridge was washed again with acidified pure water to replace the last remaining inorganic ions from the SPE cartridge. After washing, the cartridge was dried under high purity grade nitrogen gas and eluted with methanol.

Ultra-high-resolution mass spectra were acquired on a Bruker (Bremen, Germany) APEX 12 Qe Fourier transform ion cyclotron resonance mass spectrometer equipped with a 12 T superconducting magnet and a APOLLO II electrospray source. The SPE-DOM samples were diluted using methanol and introduced into the micro electrospray source at a flow-rate of 120 ml h^−1^ with a nebulizer gas pressure of 20 psi (138 kPa) and a drying gas pressure of 15 psi (103 kPa) at 250 °C through an Agilent sprayer. Spectra were externally calibrated on clusters of arginine (5 mg l^−1^ in methanol) and systematically internally calibrated with appropriate reference mass list reaching accuracy values lower than 100 ppb in routine day-to-day measurements. Data acquisition was performed using DATAANALYSIS associated software (Bruker Daltonics, version 4.0). The possible elemental formulas were calculated from the exported masses list for each peak in batch mode by a software tool written in-house (NETCALC). Final molecular formula assignments were branded into groups containing CHO, CHNO, CHOS or CHNOS molecular compositions, which were used to reconstruct the group-selective mass spectra.

### Quantitation of a_w_ and chaotropic activity

The water activity of all *Hephaestus* samples of the interface and the brine was determined empirically using a Novasina Humidat-IC-II water-activity machine fitted with an alcohol-resistant humidity sensor and eVALC alcohol filter (Novasina, Pfäffikon, Switzerland), as described previously^14,38^. The instrument was calibrated between each measurement using the pristine *Discovery* and *Kryos* brine with known water activity^10,11^. Water-activity measurements were determined at 15.0 °C three times, and variations were within ±0.002. For quantification of chaotropic activity, agar gel-points were determined by agar gelation method over a range of interface and brine concentrations using a Cecil E2501 spectrophotometer fitted with a thermoelectrically controlled heating block (Milton Technical Centre, Cambridge, England) as described previously^16^.

### Nucleic acid purification and following analysis

For DNA/RNA extraction, 2–5 l of the fractionated interface and brine samples were filtered through sterile Sterivex capsules (0.2μm pore size, Millipore) using a peristaltic pump. After filtration, filters were treated with 400 μl of TE buffer (pH 8.0) containing lysozyme (5 mg ml^−1^), vortexed for 5 sec and incubated 10 min at room temperature. 1600μl of lysis buffer QRL1 (containing β-mercaptoethanol) were added and Sterivex filters were than stored at −20 °C until processing. Total DNA and RNA were extracted using Qiagen RNA/DNA Mini Kit (Qiagen, Milan, Italy). The extraction was carried out according to the manufacturer’s instructions. DNA and RNA samples were examined by agarose gel electrophoresis and concentrations were determined using the NanoDrop ND-1000 Spectrophotometer (Wilmington, DE, USA). RNA-containing extracts were purified from DNA by Turbo DNA-free kit (Ambion, Austin, TX, USA). Each RNA sample was immediately converted into cDNA with SuperScript II Reverse Transcriptase (Invitrogen, Carlsbad, CA, USA) and hexa-random primers according to the manufacturer instructions.

Bacterial and archaeal 16S *rRNA* and key genes involved in ammonium oxidation (*amo*A), sulphur respiration (*dsr*AB), methano- (*mcr*A) and acetogenesis (*cdh*D), were amplified by PCR using primers listed in the Supplemental Table 1. All reactions were carried out in a MasterCycler 5331 Gradient PCR (Eppendorf, Hamburg, Germany). The conditions for PCR and cloning were performed as described elsewhere^10,35,39^. Positive clones from each library were randomly selected by PCR amplification. The PCR products (683 archaeal and 298 bacterial riboclones and 155 mRNA transcripts in total) were further purified and sequenced at Macrogen (Amsterdam, Netherlands). Pintail software (Ashelford *et al*., 2005) was used to checked sequences for possible chimeric origin. All sequences from 16S crDNA clone libraries were processed by the NGS analysis pipeline of the SILVA rRNA gene database project (SILVAngs 1.3)^40^. Each sequence was aligned using the SILVA Incremental Aligner (SINA v1.2.10 for ARB SVN (revision 2018)^41^ against the SILVA SSU rRNA SEED and quality controlled database. The classification was performed by a local nucleotide BLAST search against the non-redundant version of the SILVA SSU Ref dataset (release 132 (Dec 13, 2017); http://www.arb-silva.de) using blastn (version 2.2.30+; http://blast.ncbi.nlm.nih.gov/Blast.cgi) with standard settings^42^. After alignment, the neighbor-joining algorithm of ARB and MEGA 5 program packages were used to generate the phylogenetic trees based on distance analysis for 16S rRNA and functional genes, respectively. The robustness of inferred topologies was tested by bootstrap re-sampling using the same distance model (1,000 replicates).

### Nucleotide sequence accession numbers

The nucleotide sequences produced in the present study have been deposited in the DDBJ/EMBL/GenBank databases under accession numbers: MH556817 to MH556828 for archaeal *amoA* gene sequences, MH556829 to MH556850 for the bacterial *dsr*A gene sequences, MH556851 to MH556857 for the archaeal *mcr*A gene sequence and MH556858 to MH556864 for bacterial *cdh*D gene sequences.

## Supplementary information

Supplementary Info
