# Author Correction: The discovery of Lake *Hephaestus*, the youngest athalassohaline deep-sea formation on Earth

**DOI:** 10.1038/s41598-019-44367-0

**Published:** 2019-05-24

**Authors:** Violetta La Cono, Giovanni Bortoluzzi, Enzo Messina, Gina La Spada, Francesco Smedile, Laura Giuliano, Mireno Borghini, Christine Stumpp, Philippe Schmitt-Kopplin, Mourad Harir, William K. O’Neill, John E. Hallsworth, Michail Yakimov

**Affiliations:** 1 CNR, Institute for Coastal Marine Environment, Messina, 98122 Italy; 2 CNR, Institute for Marine Sciences, Bologna, 40129 Italy; 3 Mediterranean Science Commission (CIESM), MC, 98000 Monaco, Monaco; 4 CNR, Institute for Marine Sciences, La Spezia, 19136 Italy; 5 Institute of Groundwater Ecology, Helmholtz Centre Munich, Neuherberg, 85764 Germany; 6 Institute of Hydraulics and Rural Water Management, University of Natural Resources and Life Sciences Vienna, Wien, 1190 Austria; 7 Research Unit Analytical BioGeoChemistry, Helmholtz Centre Munich, Neuherberg, 85764 Germany; 8 Technische Universität München, Lehrstuhl für Analytische Lebensmittelchemie, Freising, 85354 Germany; 9 Institute for Global Food Security, School of Biological Sciences, MBC, Queen’s University Belfast, Belfast, BT9 7BL Northern Ireland UK; 10 Institute of Living Systems, Immanuel Kant Baltic Federal University, Kaliningrad, 236016 Russia

Correction to: *Scientific Reports* 10.1038/s41598-018-38444-z, published online 08 February 2019

The original version of this Article contained an error in Affiliation 7, which was incorrectly given as ‘Research Unit Environmental Simulation, Helmholtz Centre Munich, Neuherberg, 85764, Germany’. The correct affiliation is listed below:

Research Unit Analytical BioGeoChemistry, Helmholtz Centre Munich, Neuherberg, 85764, Germany

This error has now been corrected in the HTML and PDF versions of this Article.

