# Supplementary material for: The discovery of Lake Hephaestus, the youngest athalassohaline deep-sea formation on Earth
Source: Sci Rep. 2019 Feb 8;9:1679. doi: 10.1038/s41598-018-38444-z (PMC6368551; doi:10.1038/s41598-018-38444-z)
Supplement: Supplementary file 1 — Supplementary Info [file 41598_2018_38444_MOESM1_ESM.docx]

**SUPPLEMENTAL INFORMATION**

**The discovery of deep-sea Lake *Hephaestus*, the youngest magnesium-saturated athalassohaline formation on Earth.**

Violetta La Cono, Giovanni Bortoluzzi, Enzo Messina, Gina La Spada, Francesco Smedile, Laura Giuliano, Mireno Borghini, Christine Stumpp, Philippe Schmitt-Kopplin, Mourad Harir, William K. O’Neill, John E. Hallsworth and Michail Yakimov

**CONTENTS**

**Supplemental Tables 2**

**Supplemental Figures 4**

**Supplemental Table 1.** Primers and conditions used for RT-PCR amplification of functional genes.

| **Gene** | **Primer** | **Sequence (5’-3’)** | **Annealing Temperat.** | **Number**  **of cycles** | **Amplicon length (bp)** | **Reference** |
| --- | --- | --- | --- | --- | --- | --- |
| **B16S** | 530F  1492R | GCCAGCAGCCGCGGTAATAC  GGTTACCTTGTTACGACTT | 50 | 35 | 962 | Lane, 1991 |
| **A16S** | A20F  915modR | TTCCGGTTGATCCYGCCRG  GTGCTCCCCCGCCAATTYCT | 59 | 35 | 895 | De Long, 1992 |
| **CDHD** | CDH359-F  CDH837-R | GCKGAAACWGTRGAAGAMGTA  TCWCCCCAMYCKGGCWTTTC | 59°C | 35 | 478 | Present study |
| **MCRA** | mcr52-F  mcr456-R | GCTGCATACACCAACAAYAT  CTTCRTCGGAYTGGWARGAG | 53°C | 35 | 404 | Hallsworth et. al, 2007 |
| **DSRA** | DSRA97-F  DSRA921-R | TGGGABTTCCGSGGCAGCGG  TCTGGAAKCCCTGHCGCWTVAT | 65°C | 35 | 824 | Present study |
| **AMOA** | Arch-amoAF  Arch-amoAR | STAATGGTCTGGCTTAGACG  GCGGCCATCCATCTGTATGT | 55°C | 35 | 600 | Francis et al., 2005 |

Abbreviation used: B16S, bacterial 16S rRNA, A16S, archaeal 16S rRNA; CDHD, acetyl-CoA decarbonylase/synthase subunit delta; MCRA, methyl coenzyme M alpha reductase subunit alpha; DSRA, dissimilatory sulfite reductase subunit alpha; AMOA, ammonia monooxygenase subunit alpha.

**Supplemental Table 2.** Genus- and cluster-level attribution of *Hephaestus* riboclones, affiliated to *Bacteroidetes*, Gamma- and Deltaproteobacteria, is shown as a percentage of all 16S crDNA clones analyzed in corresponding library.

|  | | ***Seawater*** | ***UIF*** | ***MIF*** | **2.97M** |
| --- | --- | --- | --- | --- | --- |
|  | |  |  |  |  |
| ***Bacteriodetes*** | | |  |  |  |
| *Flavobacteriales* | 1.3 | | 0 | 0 | 0 |
| *Tangfeifania* | 0 | | 0 | 2.2 | 1.5 |
| E6aC02 clade | 0 | | 0 | 21.7 | 11.6 |
| BD2-2 clade | 0 | | 1 | 3.3 | 1.5 |
| **GammaPB** | | | | | |
| *Methylococcales* IheB2-23 | 0 | | 0 | 0 | 4.4 |
| *Chromatiales* | 0 | | 1.75 | 1.1 | 2.9 |
| *Methylococcales* ET-SHO | 0 | | 0 | 0 | 2.9 |
| *Ectothiorhodospirales* | 0 | | 0 | 0 | 1.5 |
| UBA19353 group | 7.5 | | 0 | 0 | 0 |
| *Coxiellales* | 0 | | 1.75 | 0 | 0 |
| ERP3968-O8a-Bc78 | 2.5 | | 0 | 0 | 0 |
| *Piscirickettsiales* | 1.3 | | 0 | 0 | 0 |
| *Moritella* | 1.3 | | 0 | 0 | 0 |
| SUP05 cluster | 1.3 | | 3.5 | 2.2 | 5.8 |
| *Thiomicrospira*_1 | 1.3 | | 0 | 1.1 | 1.5 |
| *Alteromonadales* | 0 | | 2 | 1.1 | 0 |
| **DeltaPB** | | | | | |
| P3OB-42 clade | | 1.3 | 0 | 0 | 0 |
| SAR324 clade | | 1.3 | 0 | 1.1 | 0 |
| OM27 clade | | 0 | 0 | 0 | 1.5 |
| *Desulfohalobium* | | 0 | 7 | 8.7 | 5.8 |
| *Desulfotignum* | | 0 | 0 | 1.1 | 0 |
| *Desulfuromonadales* | | 0 | 3.5 | 0 | 1.5 |
| PB19 clade | | 1.3 | 0 | 0 | 0 |
|  | |  |  |  |  |

**
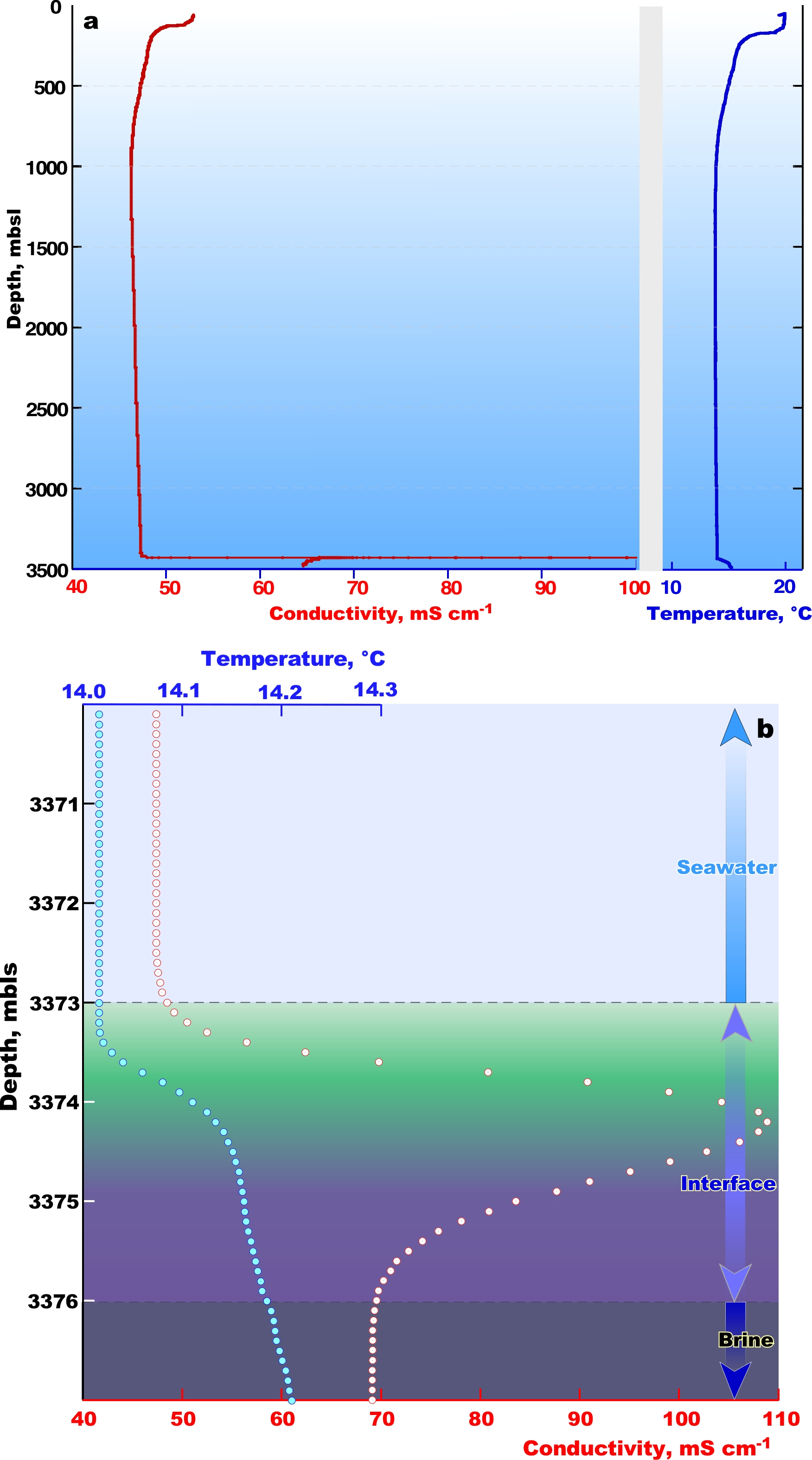
**

**Supplementary Fig. 1: The CTD profile of the whole seawater column over the Lake *Hephaestus* at sampling station S1 (a) and detailed profiles of conductivity and temperature data defining the exact depth location of the interface and brine of the lake (b).**

CTD profile, salinity data (blue) and temperature data (red) at the interface and at the bottom of

the anoxic lake

As far as all conventional online CTD sensors are calibrated for seawater and other thalassohaline hydrological formations, they are not fully functional in athalassohaline environments, dominated by bivalent cations. Therefore, the CTD profiling was used only to qualitatively localize the depth and thickness of the Hephaestus interface, while chemical analyses were done further in on-land laboraroty.


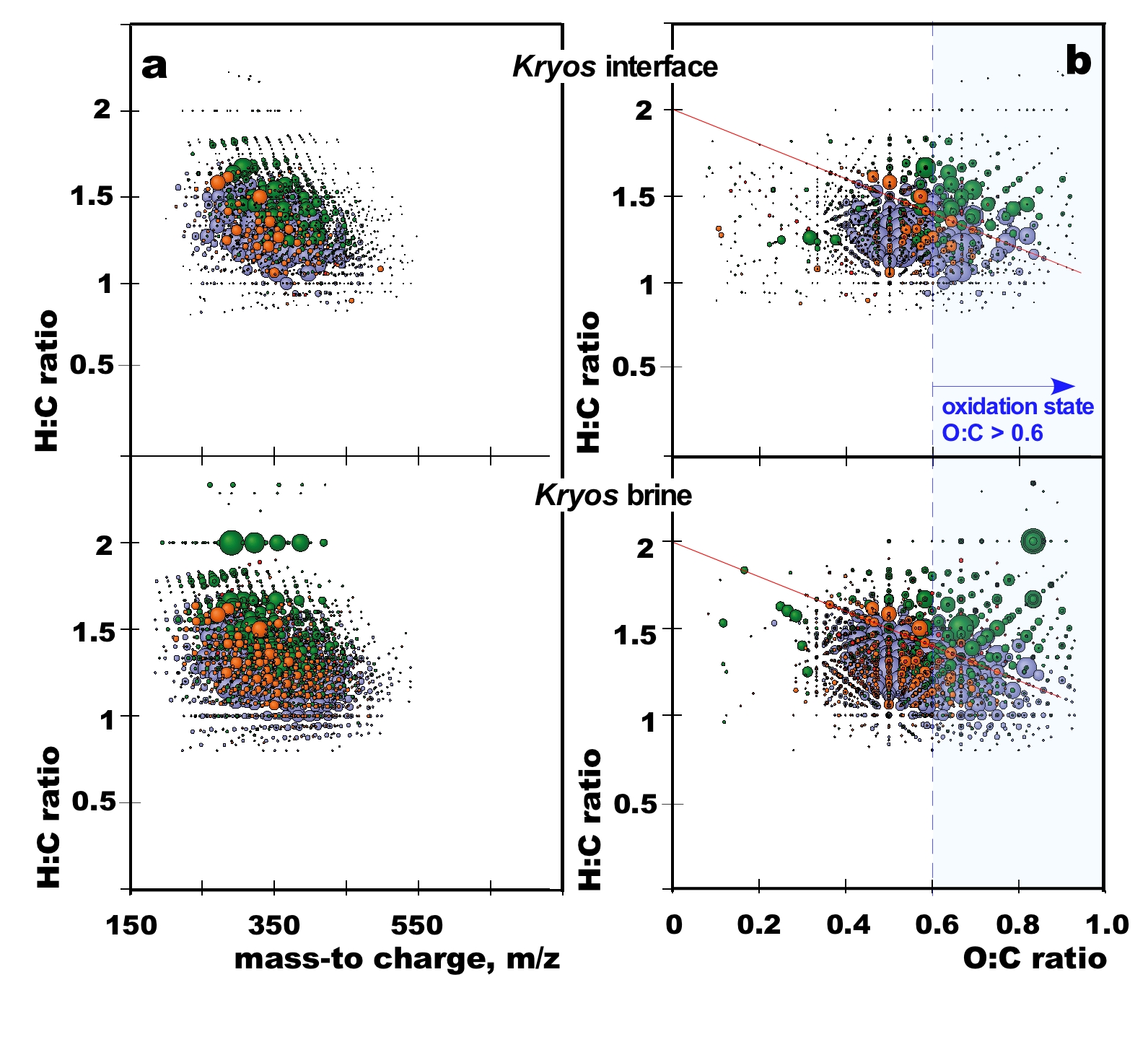


**Supplementary Fig. 2: Ultra-high resolution negative ICR-MS mass spectrometry of the *Kryos* interface and brine DOM.**

**a**, mass-to-charge versus H:C ratio, showing hundreds of low molecular weight organic compounds. **b**, van Krevelen diagrams, illustrating the high proportion of highly oxygenated (O:C ratio > 0.6) molecular series. The red line refers to the compositional range of carboxyl-rich alicyclic compounds. Bubble areas indicate relative mass peak intensity of each assigned mass ion. Bubble color code for molecular formula series with C, H, O, N and S combinations are defined as follow: CHO (blue), CHOS (green), CHON (orange) and CHONS (red).

**
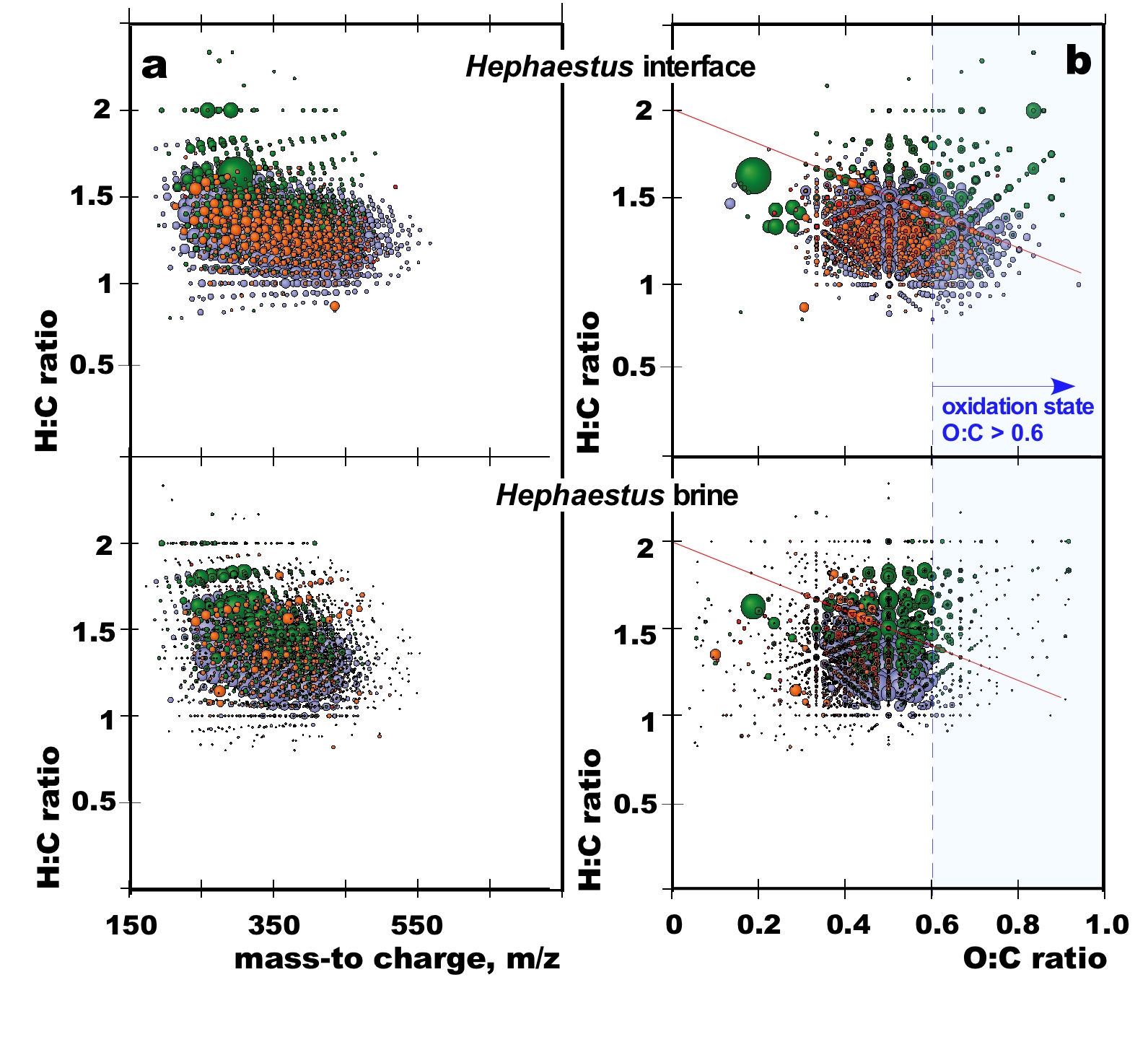
**

**Supplementary Fig. 3: Ultra-high resolution negative ICR-MS mass spectrometry of the *Hephaestus* interface and brine DOM.**

**a**, mass-to-charge versus H:C ratio, showing hundreds of low molecular weight organic compounds. **b**, van Krevelen diagrams, illustrating remarkable depletion of highly oxygenated (O:C > 0.6) molecular series in the *Hephaestus* brine DOM compared to those of the interface. The red line refers to the compositional range of carboxyl-rich alicyclic compounds. Bubble areas indicate relative mass peak intensity of each assigned mass ion. Bubble color code for molecular formula series with C, H, O, N and S combinations are defined as follow: CHO (blue), CHOS (green), CHON (orange) and CHONS (red).

**
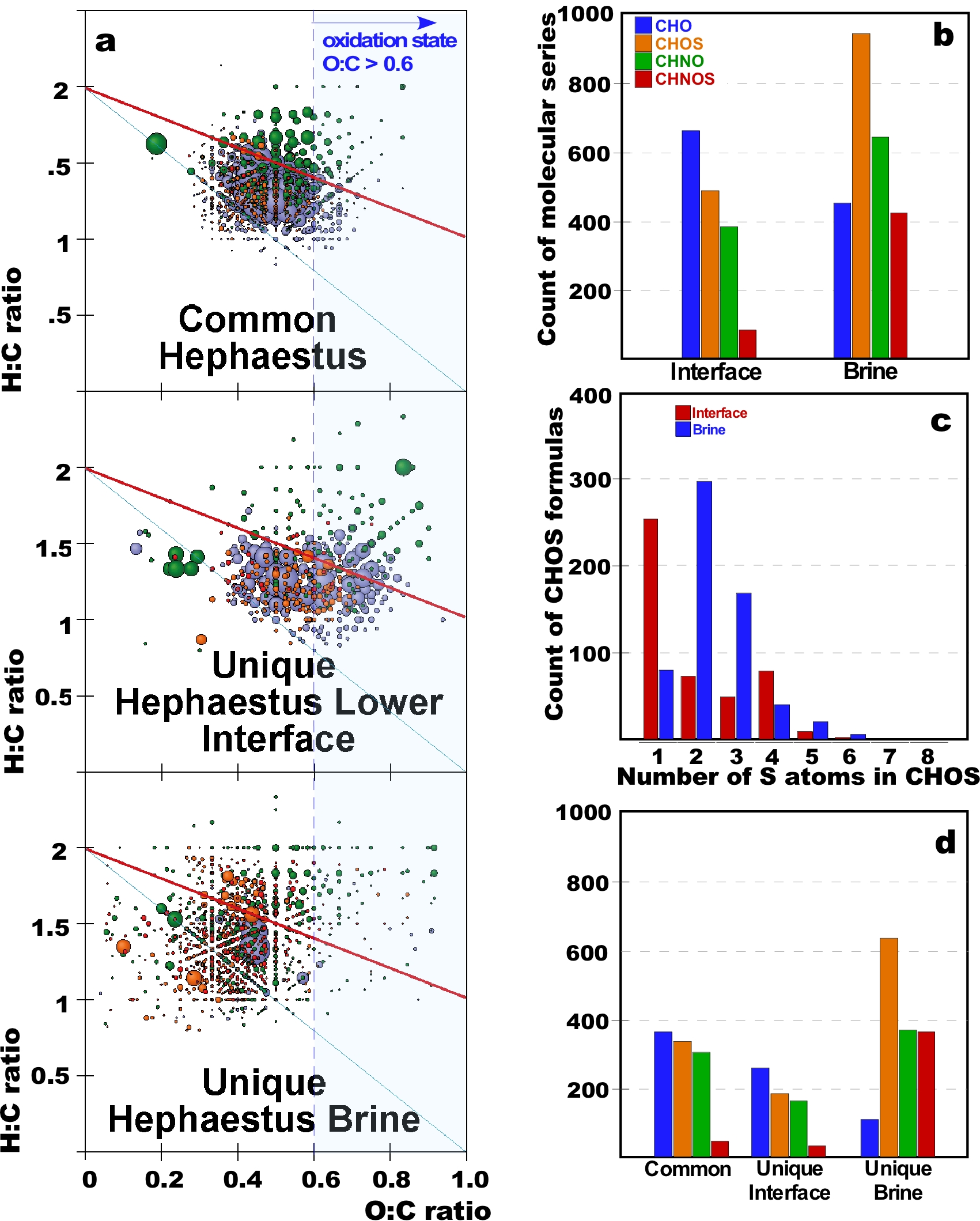
**

**Supplementary Fig. 4:** **Unique and common molecular formulas derived from detected mass ions in the brine and interface samples of Lake *Hephaestus*.**

**a**, van Krevelen diagrams, illustrating remarkable depletion of common highly oxygenated (O:C > 0.6) molecular series in *Hephaestus* brine compared with interface. The red line refers to the compositional range of carboxyl-rich alicyclic compounds and the blue line refers to any fully saturated open-chain aliphatic (poly)carboxylic acids. Bubble color code for molecular formula series with C, H, O, N and S combinations are defined as follow: CHO (blue), CHOS (green), CHON (orange) and CHONS (red). Bubble areas indicate relative mass peak intensity of each assigned mass ion. **b**, counts of elemental compositions of assigned molecular formulas CHO, CHNO, CHOS and CHNOS. **c**, counts of sulfur atoms in CHOS-containing compounds. **d**, hiistogram representing relative counts of respective unique and common CHO (blue), CHOS (orange), CHNO (green) and CHNOS (red) molecular series as found in respective van Krevelen diagrams.

**
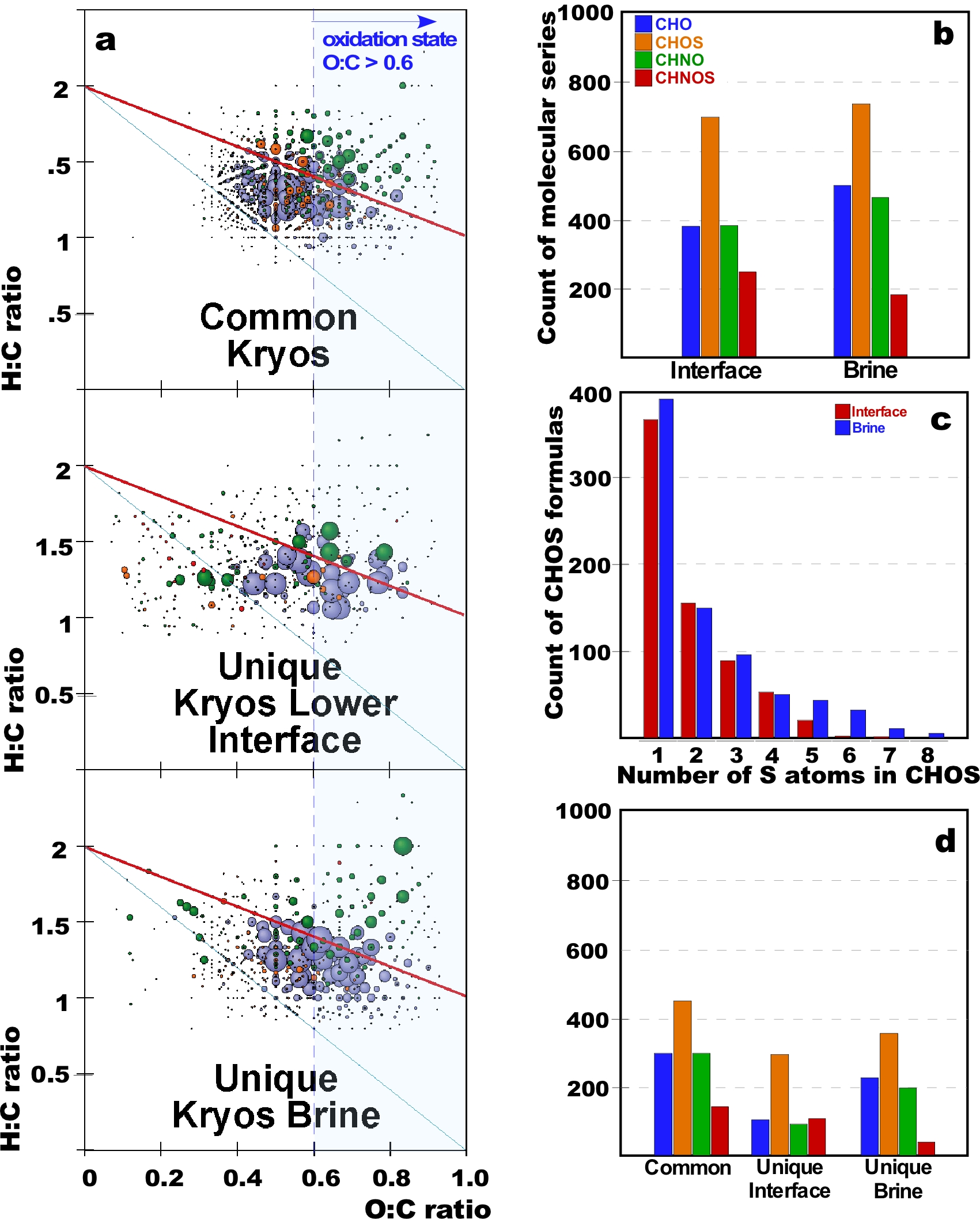
**

**Supplementary Fig. 5:** **Unique and common molecular formulas derived from detected mass ions in the brine and interface samples of Lake *Kryos*.**

**a**, van Krevelen diagrams, illustrating the high proportion of highly oxygenated (O:C ratio > 0.6) molecular series in *Kryos* samples. The red line refers to the compositional range of carboxyl-rich alicyclic materials and the blue line refers to any fully saturated open-chain aliphatic (poly)carboxylic acids. Bubble color code for molecular formula series with C, H, O, N and S combinations are defined as follow: CHO (blue), CHOS (green), CHON (orange) and CHONS (red). Bubble areas indicate relative mass peak intensity of each assigned mass ion. **b**, counts of elemental compositions of assigned molecular formulas CHO, CHNO, CHOS and CHNOS. **c**, counts of sulfur atoms in CHOS-containing compounds. **d**, histogram representing relative counts of respective unique and common CHO (blue), CHOS (orange), CHNO (green) and CHNOS (red) molecular series as found in respective van Krevelen diagrams.


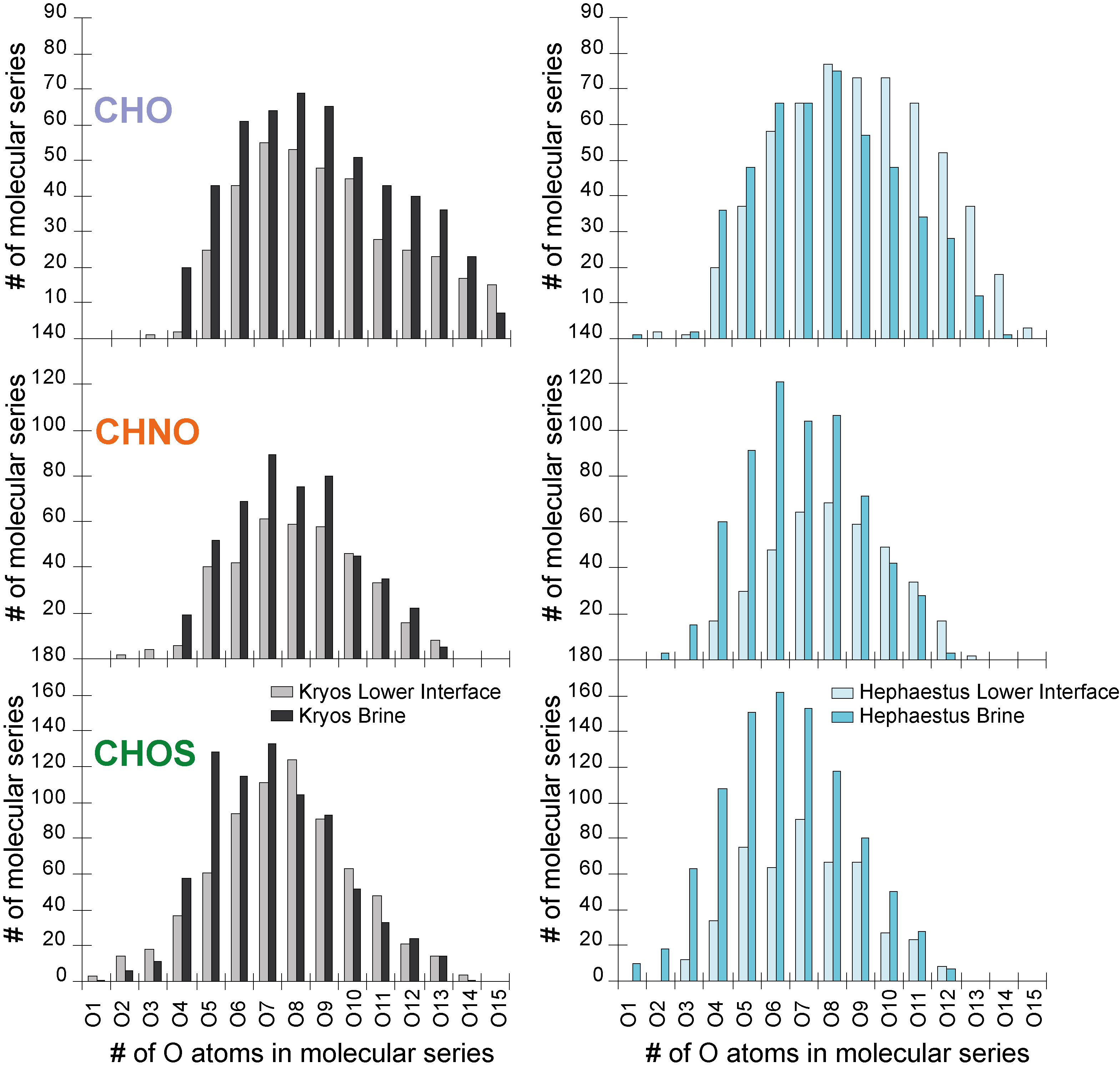


**Supplementary Fig. 6: Counts of oxygen atoms involved in CHO, CHNO and CHOS molecular series in brine and interface samples of the lakes *Kryos* and *Hephaestus*.**


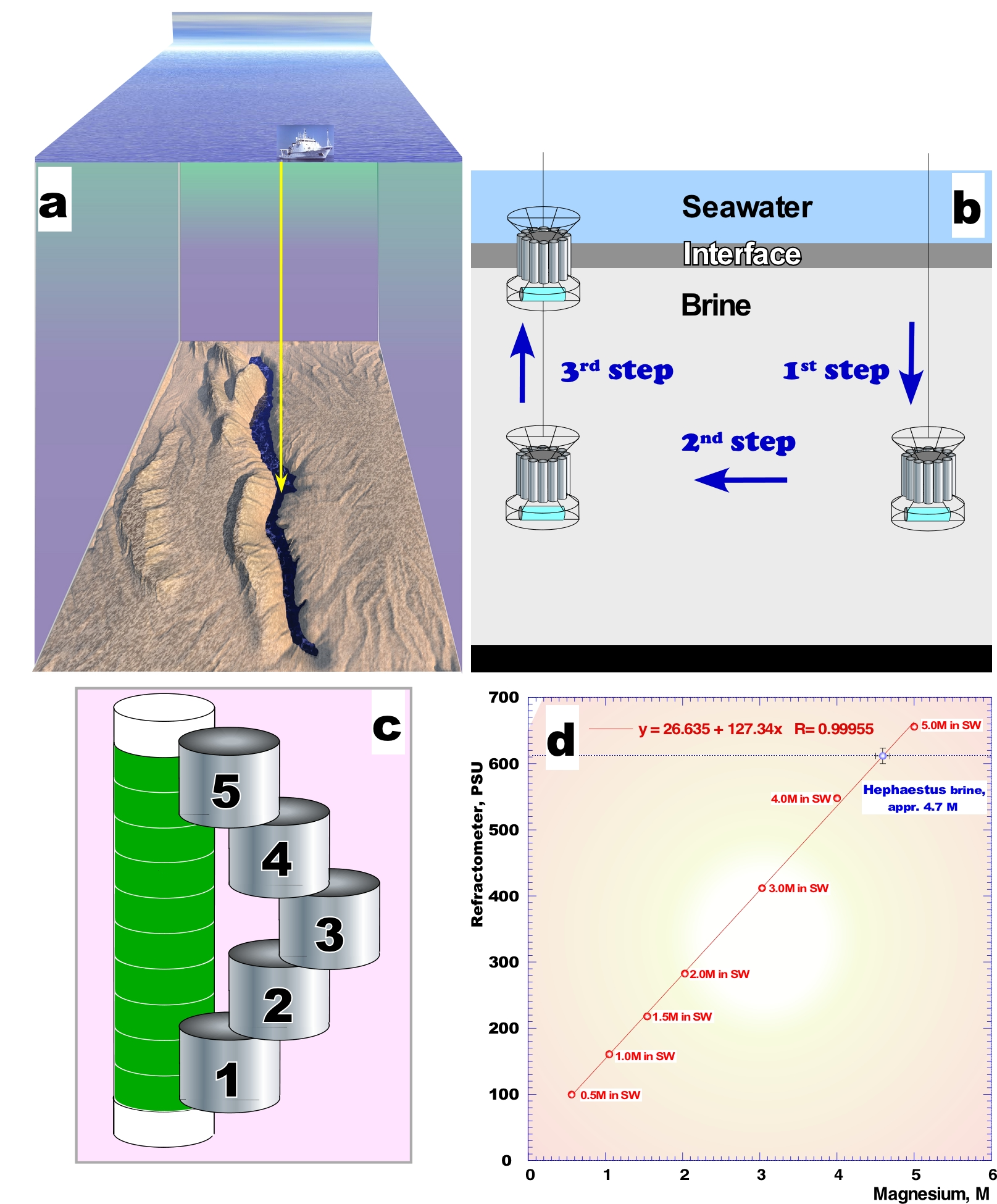


**Supplementary Fig. 7: High-resolution sampling strategy.**

During the cruises DEEPPRESSURE_2013 and SALINE_2014, the interface was captured and fractionated as described elsewhere up to 10 cm-scale resolution sampling^7,22^. Briefly, during bottom-wards moving at velocity of 0.3-0.6 m s^-1^, the CTD sensor, housed on a rosette with 18×12 L Niskin bottles, indicated the exact vertical positioning of the interface by large increase in conductivity (**a**). The rosette continued to move down into the brine for additional 25 meters and than stopped to straight the released cable and to stabilize the vertical positioning of rosette (**b**, 1^st^ step). During the second phase of sampling, ROV *Urania* drifted 50-100 m away from the site where rosette has entered into the brine lake (**b**, 2^nd^ step). In the final phase, the rosette was slowly recovered from the brine (<0.1 m s^-1^) and the Niskin bottles were closed at the depth, corresponding the established positioning of the interface (**b**, 3^rd^ step). After cast recovery, the captured interface was sub-sampled into one-liter fractions (**c**). The top-most and bottom-most fractions of the Niskin bottles were disregarded for further analyses. Using a hand refractometer (Atago, Tokyo, Japan), the salinity of each subsample was monitored and obtained values were plotted against the standard curve of MgCl_2_ solutions in seawater (**d**).

**
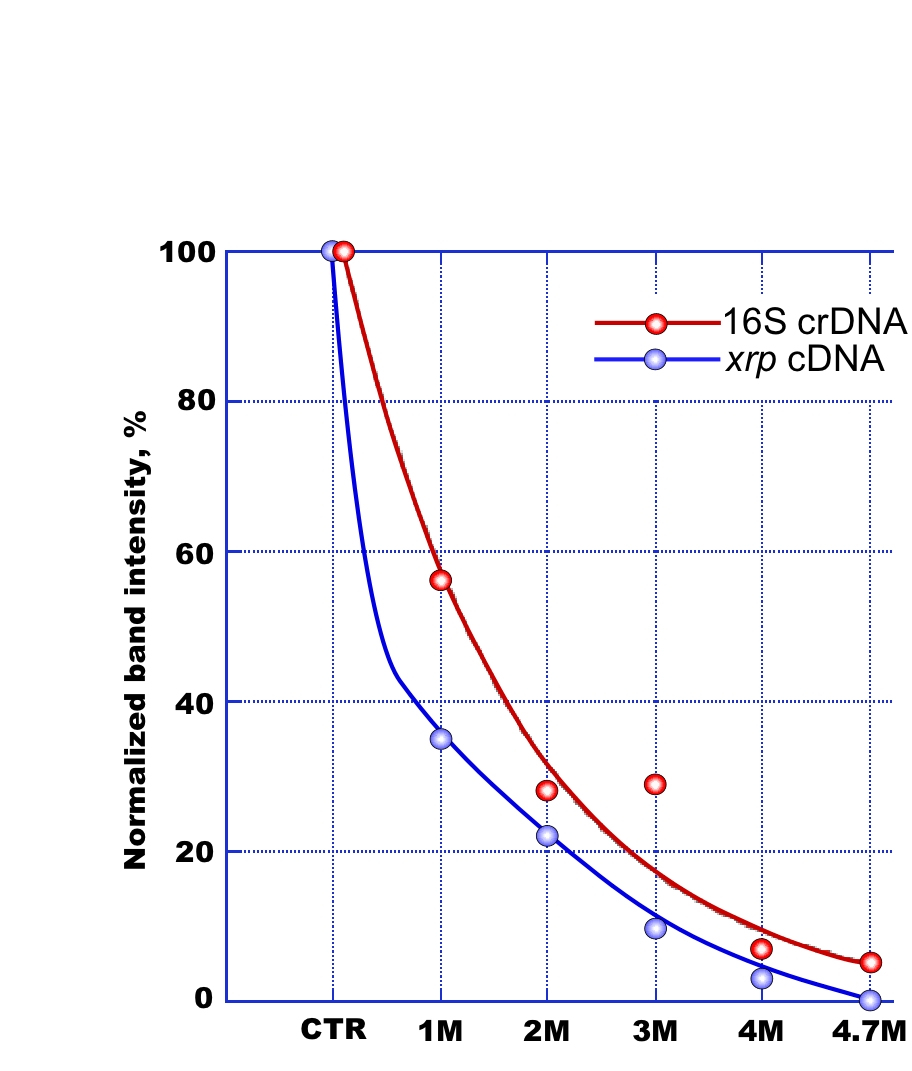
**

**Supplementary Fig. 8: Rapid degradation of mRNA (xenorhodopsin transcripts) and 16S rRNA in high-magnesium brines**.

The rapid degradation of *xrp* mRNA (*Nanohaloarchaea* xenorhodopsin) and 16S rRNA transcripts in the high-magnesium brines were confirmed in a simulation experiment in which *Nanohaloarchaea*-containing enrichment, obtained from the seawater/*Hephaestus* brine interphase, were incubated during 24 hours in the artificially prepared brines with increasing concentrations of MgCl_2_ (1.0 – 4.7 M). DNA was only moderately degraded after 24 hours of incubation and both 16S rDNA and *xrp* gene sequences were amplified (CTR). In contrast, both types of RNA molecules were rapidly degraded and amplification of *xrp* mRNA was unsuccessful in brines with > 3.0 M of MgCl_2_. The ability to weakly amplify 16S rRNA in 4.0 and 4.7 M MgCl_2_ brines, can be explained by not only inherently higher stability of rRNA compared with mRNA, but also by the much higher cellular concentrations of the former, because the ratio of 16S rRNA to a particular species of mRNA is greater than 1000:1. Chaotropic salts have been shown to kill (Duda *et al*., 2004) but, like MgCl_2_ at high concentrations, to preserve dead cells in a form that may, when sampled and analysed by some currently used methods (DNA and lipids), be interpreted as alive.


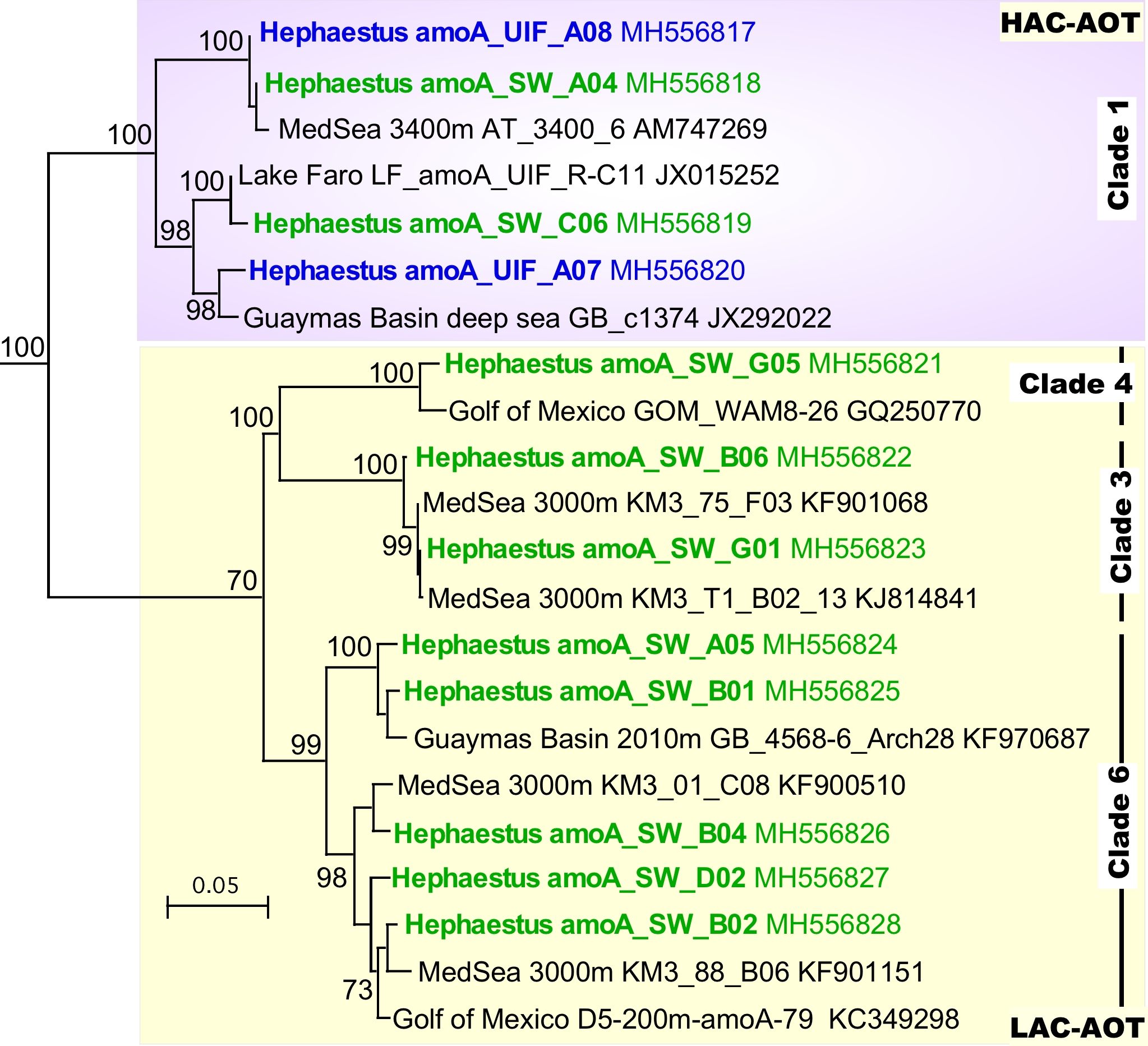


**Supplementary Fig. 9: Neighbor-joining phylogenetic nucleotide tree of thaumarchaeotal *amoA* recovered from the *Hephaestus* upper interface (UIF) and deep seawater from 3,372 mbsl (SW).**

The marine clade 1 of high ammonia concentration ammonium-oxidizing archaea (HAC-AOT) and the marine clades 3,4 and 6 of low ammonia concentration ammonium-oxidizing archaea (LAC-AOT), as defined by Sintes et al. (2016), are color-coded as magenta and yellow, respectively. Sequences of clones are also color-coded according to types of clone library origins, as green (SW) and blue (UIF). Significant bootstrap values (>70) are shown at branch nodes. The *amoA*-phylogenetic tree was rooted with *Cenarchaeum symbiosum A* (DQ397580). The scale bar represents the number of fixed mutations per nucleotide position.


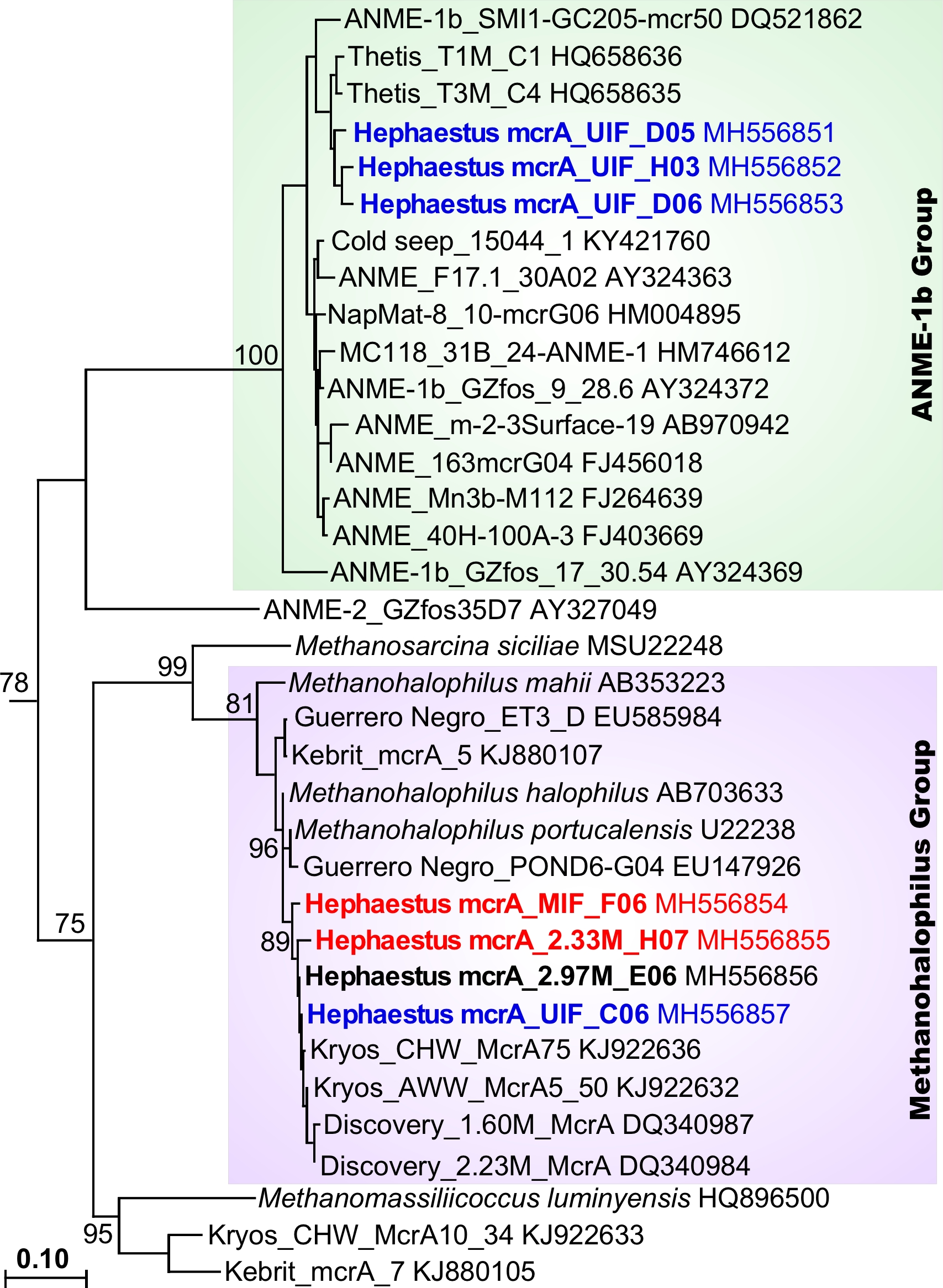


**Supplementary Fig. 10: Phylogenetic analyses of *mcr*A gene transcripts recovered from the *Hephaestus* interface.**

Analysis of sequences derived from mRNA coding for methyl co-M reductase (*mcr*A) indicates that methanotrophic group ANME-1b is active only in the upper interface (UIF) layer, while methanogenic organisms affiliated to *Methanohalophilus* Group are active in all analysed layers. The numbers at the nodes indicate the percentages of recovery in 1000 bootstrap re-samplings. Sequences of recovered clones are color-coded according to Figures 3 and 4 in the main text. Only relevant bootstrap values of ≥ 70% are shown. The scale bar corresponds to 10% estimated difference in nucleotide sequence positions. The *mcr*A-phylogenetic tree was rooted with *Methanobrevibacter arboriphilus* DSM 1125 *mcr*A (AF414035) gene sequence*.*


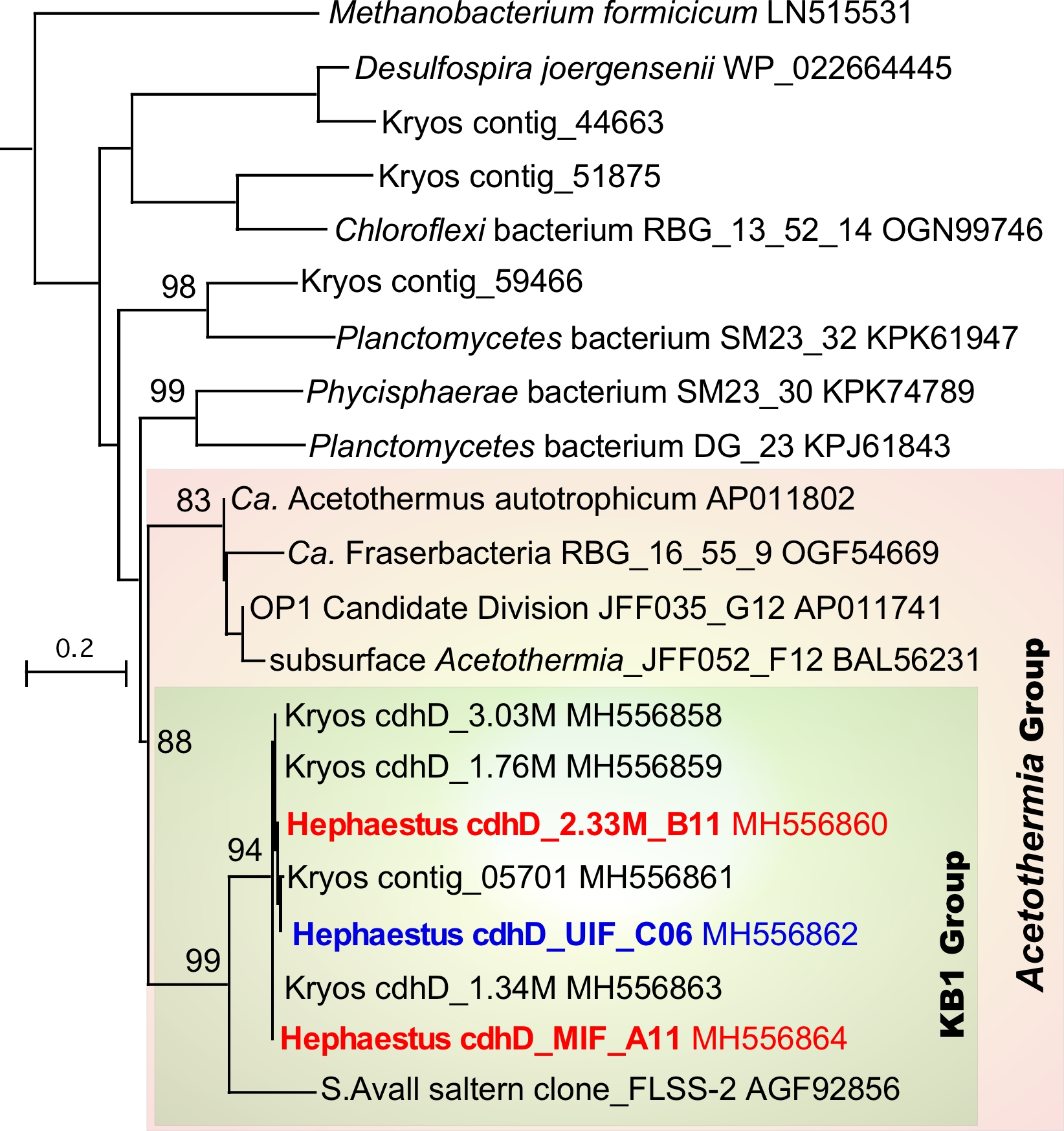


**Supplementary Fig. 11: Phylogenetic analyses of acetyl-CoA decarbonylase / synthase complex subunit delta (CdhD) gene transcripts recovered from the *Hephaestus* interface.**

Analysis of obtained sequences indicates that this monophyletic KB1 group of *Acetothermia* is active only in the upper (UIF) and middle (MIF) interface layers. The numbers at the nodes indicate the percentages of recovery in 1000 bootstrap re-samplings. Sequences of recovered *Hephaestus* clones are color-coded according to Figures 3 and 4 in the main text. Only relevant bootstrap values of ≥ 75% are shown. The scale bar corresponds to 20% estimated difference in amino acids sequence positions. The tree was rooted with acetyl-CoA decarbonylase/synthase complex subunit gamma, CdhE (OGF54456) of *Ca*. Fraserbacteria.


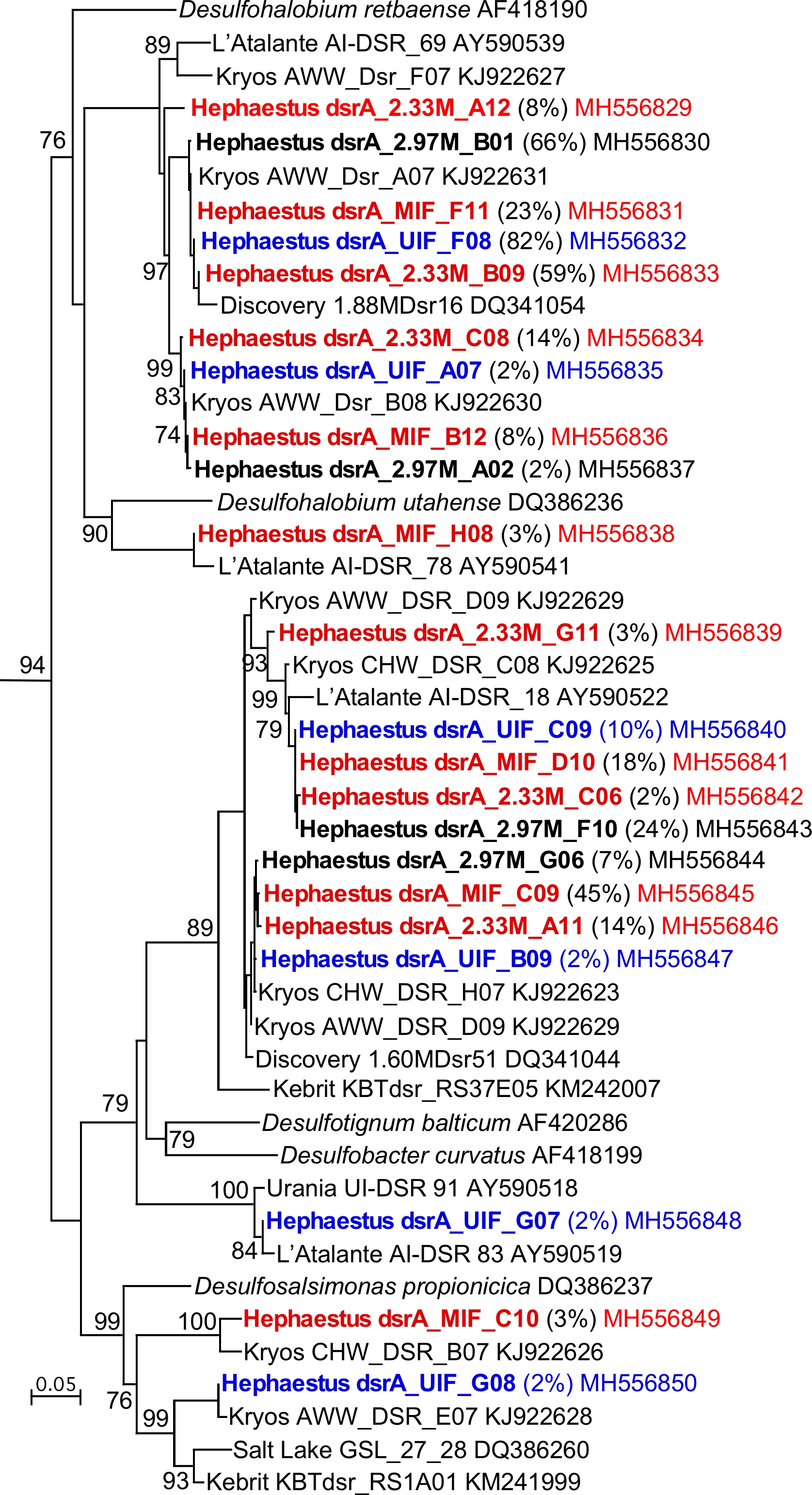


**Supplementary Fig. 12:** **Phylogenetic analysis of *dsr*A gene transcripts recovered from the *Hephaestus* interface.**

Analysis of sequences derived from mRNA coding for dissimilatory sulfite reductase subunit A (DsrA) indicates that sulfate-reducing bacteria are active in all analyzed layers of the interface. Percentage of each *dsr*A ecotype recovered in corresponding libraries shown in parenthesis. The numbers at the nodes indicate the percentages of recovery in 1000 bootstrap re-samplings. Only relevant bootstrap values of ≥ 70% are shown. Sequences of recovered clones are color-coded according to Figures 3 and 4 in the main text. The scale bar corresponds to 5% estimated difference in nucleotide sequence positions. The tree was rooted with *Thermodesulforhabdus norvegica dsr*AB (AF334597) gene sequence.
